# Seasonal patterns of influenza incidence and the influence of meteorological and air pollution factors in Thailand during 2009–2019

**DOI:** 10.1016/j.heliyon.2024.e36703

**Published:** 2024-08-22

**Authors:** Suparinthon Anupong, Charin Modchang, Sudarat Chadsuthi

**Affiliations:** a Department of Chemistry, Mahidol Wittayanusorn School (MWIT), Salaya, Nakhon Pathom, 73170, Thailand; b Biophysics Group, Department of Physics, Faculty of Science, Mahidol University, Bangkok, 10400, Thailand; c Centre of Excellence in Mathematics, MHESI, Bangkok, 10400, Thailand; d Thailand Center of Excellence in Physics, Ministry of Higher Education, Science, Research and Innovation, 328 Si Ayutthaya Road, Bangkok, 10400, Thailand; e Department of Physics, Faculty of Science, Naresuan University, Phitsanulok, 65000, Thailand

**Keywords:** Influenza, Seasonality, Wavelet analysis, Weather, Air pollution

## Abstract

Influenza, an acute respiratory illness, remains a significant public health challenge, contributing substantially to morbidity and mortality worldwide. Its seasonal prevalence exhibits diversity across regions with distinct climates. This study aimed to explore the seasonal patterns of influenza and their correlation with meteorological and air pollution factors across six regions of Thailand. We conducted an analysis of monthly average temperature, relative humidity, precipitation, PM10, NO_2_, O_3_ concentrations, and influenza incidence data from 2009 to 2019 using wavelet analysis. Our findings reveal inconsistent biannual influenza prevalence patterns throughout the study period. The biannual pattern emerged during 2010–2012 across all regions but disappeared during 2013–2016. However, post-2016, the biannual cycles resurfaced, with peaks occurring during the rainy and winter seasons in most regions, except for the southern region. Wavelet coherence reveals that relative humidity can be the main influencing factor for influenza incidence over a one-year period in the northern, northeastern, central, Bangkok-metropolitan, and eastern regions, not in the southern region during 2010–2012 and 2016–2018. Similarly, precipitation can drive the influenza incidence at the same period for the northeastern, central, Bangkok-metropolitan, and eastern regions. PM10 concentration can influence influenza incidence over a half-year period in the northeastern, central, Bangkok-metropolitan, and eastern regions of Thailand during certain years. These results enhance our understanding of the temporal dynamics of influenza seasonality influenced by weather conditions and air pollution over the past 11 years. Such knowledge is invaluable for resource allocation in clinical settings and informing public health strategies, particularly in navigating Thailand's climatic complexities.

## 1 Introduction

Influenza is an acute respiratory disease that remains a major public health concern and contributes significantly to both morbidity and mortality [1]. Globally, between 1995 and 2015, seasonal influenza-associated respiratory illnesses were estimated to cause 290,000 to 650,000 deaths annually [2]. In 2019, the Global Burden of Disease Study (GBD) 2017 estimated 145,000 (95 % uncertainty interval: 99,000–200,000) deaths from influenza lower respiratory tract infections [3]. The occurrence of the pandemic leads to an increase in the burden of influenza. Before the 2009 influenza pandemic, influenza caused 148,000–249,000 respiratory deaths annually [4], compared to an estimated 152,000–575,000 deaths from the (H1N1)pdm09 virus infection during the first 12 months of 2009, according to Dawood et al. [5].

In the tropics and subtropics, many studies suggest that the burden of influenza is not notably lower than in temperate regions [[6], [7], [8]]. The seasonality of influenza varies with latitude throughout the Northern and Southern hemispheres based on the study in the Americas [9,10]. In temperate regions, influenza peaks in winter and is less prevalence during summer [8,10]; however, seasonality varies in the tropics [6]. In some countries, influenza epidemics occur during the rainy season, while in others, influenza presents year-round with multiple peaks or no clear seasonal pattern [6,11].

Climate factors are suggested to be the main drivers for the seasonal pattern of influenza [12]. The study of Tamerius suggests that cold-dry conditions are associated with seasonality in temperate regions, whereas humid-rainy condition contribute to the seasonal patterns in tropical regions [13]. In temperate regions, a study in the Netherlands found a negative relationship between specific humidity and influenza incidence from 2015 to 2019 [14]. In the Republic of Korea, low daily temperatures (0–5 °C) and low (30%–40 %) or high (70 %) relative humidity increased the risk of influenza incidence [15]. In tropical and subtropical regions, the role of temperature and humidity remains unclear [12]. A study in three tropical Central American countries found a positive association with humidity in El Salvador and Panama but not in Guatemala [16]. In French Guiana, low specific humidity and increased rainfall were linked to higher influenza incidence [17]. Whereas in Kenya, low temperatures and low specific humidity were significantly associated with increased influenza activity, with no significant association with rainfall [18].

The association between air pollution and influenza has also become an important for public health concern. A study in Warsaw, Poland, found that influenza-like illnesses (ILI) morbidity increased with rising PM2.5 and PM10 concentrations [19]. A retrospective study in China from 2004 to 2017 found a significant positive correlation between influenza incidence and air pollutants (PM2.5, PM10, SO_2_, NO_2_, and CO) [20]. Another study based on provincial-level surveillance data in China from 2013 to 2018 found O_3_ to be a driver of the influenza transmissibility [21]. Exposure to air pollutants can impair immune responses and affect the host's immunity to respiratory virus infections [22]. Particulate matter (PM) of small size (PM10, PM2.5, and PM1) has negative health impacts, both in terms of mortality and adverse health effects, as it causes inflammation and oxidative stress, compromising pulmonary immunity and increasing the susceptibility to infection. Even relatively small changes in PM can significantly impact on human health and mortality [23].

In Thailand, there have been a few studies on the effects of meteorological factors and air pollutants on influenza transmission. One study in the central and southern regions of Thailand found a correlation between the average minimum relative humidity and influenza cases [24]. This study also highlighted the significance of average temperatures in influencing influenza transmission in the southern region. Another study in Bangkok suggested a direct relationship between heightened relative humidity levels and increased influenza activity [11]. The seasonal pattern of influenza cases in Thailand exhibited a bimodal distribution, with two annual peaks occurring in February and between August and September. Conversely, a study in Chiang Mai Northern Thailand, conducted from 2011 to 2020, revealed a pronounced seasonal pattern of influenza cases aligning with the colder months of January and February [25]. This study also found that PM2.5 and a one-month lag in temperature were associated with influenza incidence. Recently, a retrospective study in Bangkok during 2018–2020 found that high levels of PM2.5 were associated with the number of upper respiratory infections [26].

Prior investigations have notably lacked a comprehensive study of air pollutants and meteorological variables across Thailand. Thus, understanding the influence of these factors on influenza and their contributions to seasonal patterns is essential for formulating effective control strategies that mitigate and manage the disease's impact. This study aimed to accurately probe the seasonality of influenza and untangle potential correlations between these factors and influenza incidences in six different regions of Thailand, with the ultimate goal of identifying underlying driving mechanisms. By employing a dataset from 2009 to 2019, we performed wavelet analysis and coherence to rigorously assess the impact of these identified risk factors on influenza incidences.

## 2 Materials and methods

### 2.1 Data collections

**Fig. 1:**
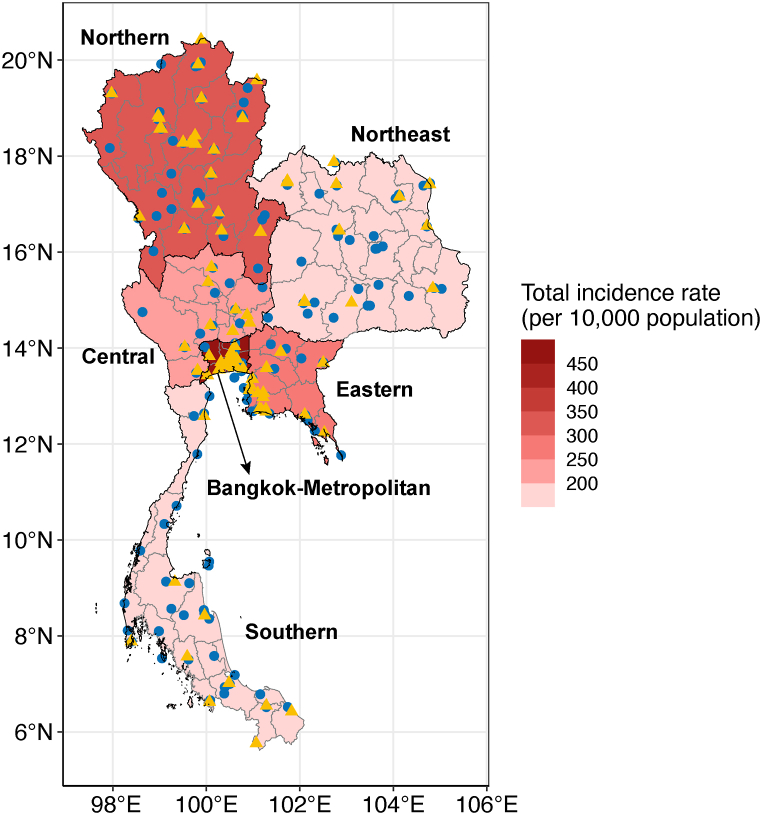
Map of Thailand showing the influenza incidence rate (total influenza cases per 10,000 population from 2009 to 2019) in six different regions: Northern, Northeastern, Central, Bangkok-Metropolitan, Eastern, and Southern. Blue circles indicate the location of weather stations, while yellow triangles indicate the location of air pollution stations. (For interpretation of the references to color in this figure legend, the reader is referred to the Web version of this article.)

Monthly influenza reported cases from 76 provinces in Thailand from 2009 to 2019 were extracted from the National Disease Surveillance (Report 506), Division of Epidemiology, Department of Disease Control, Ministry of Public Health, Thailand, which is available online [27]. The reported cases consist of suspected, probable, and confirmed cases. Most reported cases are suspected cases, classified based on clinical criteria: individuals with fever and cough, and at least one of the following symptoms: sore throat, runny nose, headache, and tiredness, according to the case definition for communicable diseases surveillance [28]. A probable case is defined as an individual who has clinical symptoms based on clinical criteria and has an epidemic history connected to confirmed cases or has a presumptive diagnosis using a rapid influenza diagnostic test. Some samples of suspected and probable cases are then tested based on the specific diagnosis, such as RT-PCR, viral isolation, or serological test for laboratory confirmation, and are defined as confirmed cases. In our study, the reported cases–including suspected, probable, and confirmed cases were summed in each of the six regions: Northern (N), Northeastern (NE), Central (C), Bangkok Metropolitan (BKK), Eastern (E), and Southern (S), classified based on the Thai meteorological department [29] (see Fig. 1). The influenza incidence rate is defined as the reported cases per 10,000 population for our analysis.

For the meteorological factors, we use daily time series provided by the Global Surface Summary of the Day (GSOD) using the R package ‘GSODR’ [30], which includes mean temperature, mean relative humidity, and mean precipitation from 2009 to 2019. The daily time series were averaged to monthly time series, and then the monthly data were averaged from all stations in each region (32, 35, 16, 18, 32, and 13 stations for N, NE, C, E, S, and BKK regions, shown as blue circles in Fig. 1).

For air pollution, the monthly time series of PM10, NO_2_, and O_3_ concentrations were provided by the Pollution Control Department, Thailand [31]. The regional data were retrieved from the average data in each region, shown as yellow triangles in Fig. 1 (24, 13, 13, 19, 17, and 33 stations for N, NE, C, E, S, and BKK regions). We applied the log transform to all variables for meteorological factors, air pollutants, and influenza incidence before applying the wavelet analysis [32].

### 2.2 Wavelet transform analysis

In this work, we use wavelet analysis, a technique that has been widely applied in various fields, including the study of infectious diseases such as dengue [33], measles [34], COVID-19 [35], and influenza [36,37]. This method can highlight the time-frequency domain for nonstationary time series and identify both positive and negative associations simultaneously. We employed four types of wavelet analyses: continuous wavelet transform (CWT), wavelet transform coherence (WTC), partial wavelet coherence (PWC), and multiple wavelet coherence (MWC). Initially, we applied CWT to study the seasonality of influenza incidence in the six regions of Thailand. We then examined their correlations with meteorological factors (temperature, relative humidity, and precipitation) and air pollution (PM10, NO_2_, and O_3_ concentrations) using WTC. To determine the partial correlation in the resulting WTC between two time series, PWC is employed to observe the impact of one variable on influenza incidence while eliminating another. Furthermore, the results are confirmed using MWC, which helps to identify the coherence of multiple independent variables on a dependent variable.

CWT provides the time-frequency domain, where the frequency changes over time using the base ‘Morlet mother wavelet’ function [38]. To investigate the connections between influenza incidence and meteorological factors, as well as between influenza incidence and air pollution, we applied WTC to search for the co-movement of two different time series, X and Y. The WTC is defined as follows:

(1) $$R^{2}=\frac{{\left|S\left(s^{-1}W_{n}^{XY}\left(s\right)\right)\right|}^{2}}{S\left(s^{-1}{\left|W_{n}^{X}\left(s\right)\right|}^{2}\right)∙S\left(s^{-1}{\left|W_{n}^{Y}\left(s\right)\right|}^{2}\right)}\text{,}$$

where the range of $R^{2}$ is between 0 and 1, indicating no coherence at 0 and perfect coherence at 1. The Monte Carlo approach is used by generating synthetic red noise data simulated from the observed data [32]. Wavelet transforms are then performed on both the synthetic and observed data to calculate wavelet power spectra. Significant regions in the observed data are identified by comparing these spectra. We can interpret the WTC as the covariance between two-time series, with contour lines indicating the consistency of their periodicities. The bi-directional (lead-lag) relationships between different time-period combinations can be captured.

Several features (meteorological factors and air pollutants) might correlate with influenza incidence. To study the effect of each feature, we used the partial wavelet coherence (PWC) focusing on one feature's effect while eliminating others, similar to partial correlation [39]. The PWC is the result of WTC between two time series Y and $X_{1}$ by removing the influence of the time series $X_{2}$. PWC squared ($RP^{2}\left(Y,X_{1},X_{2}\right)$) shows the correlation between Y and $X_{1}$, as shown in equation (2):

(2) $$RP^{2}\left(Y,X_{1},X_{2}\right)=\frac{{\left|R\left({Y,X}_{1}\right)-R\left({Y,X}_{2}\right)-{R\left(Y,X_{1}\right)}^{*}\right|}^{2}}{{\left[1-R\left({Y,X}_{2}\right)\right]}^{2}{\left[1-R\left(X_{2},X_{1}\right)\right]}^{2}}\text{.}$$

Where R represents the coherence between two time series. PWC squared (after the elimination of the effect of $X_{2}$) is determined similarly to partial correlation squared, which $RP^{2}$ ranging from 0 to 1. In the period-time domain, a position with a low value of PWC squared where there is a high value of WTC squared of Y and $X_{1}$, implies that the time series $X_{1}$ does not significantly affect time series Y, and $X_{2}$ is considered as the dominant factor influencing time series Y.

Similar to PWC, multiple wavelet coherence (MWC) works like multiple correlation, providing the wavelet coherence of time series Y depending on the linear combination of two independent time series $X_{1}$ and $X_{2}\text{.}$ MWC, $RM^{2}\text{,}$ is defined as follows [39]:

(3) $$RM^{2}\left(Y,X_{1},X_{2}\right)=\frac{R^{2}\left({Y,X}_{1}\right)+R^{2}\left({Y,X}_{2}\right)-2Re\left[R\left({Y,X}_{1}\right)∙{R\left({Y,X}_{2}\right)}^{*}∙{R\left(X_{2},X_{1}\right)}^{*}\right]}{1-R^{2}\left(X_{2},X_{1}\right)}\text{.}$$

The MWC result shows the effect of the combined two independent variables on the seasonal pattern of influenza incidence. A full description of four wavelet analyses is provided in the supplementary information.

For the wavelet analysis, we used the R package “biwavelet” v0.20.21 [40] to analyze the seasonality of influenza incidence across six regions of Thailand with CWT. Moreover, we studied the association between influenza incidence and meteorological factors (temperature, relative humidity, and precipitation), as well as the association between influenza incidence and air pollution (PM10, NO_2_, and O_3_ concentrations) by using WTC. We also investigated PWC and MWC to examine the partial and multiple correlations of meteorological factors and air pollutants influencing influenza incidence.

## 3 Results

### 3.1 Descriptive statistics

A total of 1,505,852 influenza cases, with influenza incidence (per 10,000 population) of 1,454.65, were reported in Thailand during the 11-year study period from 2009 to 2019. According to the Thai Meteorological Department [29], influenza incidences were categorized into five regions: Northern (N), Northeastern (NE), Central (C), Eastern (E), and Southern (S). We observed that the influenza incidences in Bangkok Metropolitan (BKK) significantly differed from those of the Central region (Fig. 1). Due to the notably high levels of incidence rates and population in BKK, we decided to separate BKK from the C region. The total influenza incidence rates recorded over the 11-year study period were 290, 148, 184, 432, 250, and 150 for the N, NE, C, BKK, E, and S regions, respectively (Fig. 1). A statistical description of influenza incidences for each region is shown in Table 1.

**Table 1:** Descriptive analysis of the meteorological factors, air pollutants, and influenza incidence rate of 6 regions during 2009–2019.

Region	variable	Mean	SD	Min	P25	Median	P75	Max
Northern	Incidence rate	2.20	2.12	0.07	0.72	1.45	2.87	10.82
	Temperature	26.74	2.27	20.51	25.55	27.25	28.03	32.07
	Relative Humidity	71.87	8.66	49.65	65.68	73.80	79.58	83.42
	Precipitation	3.62	3.14	0.00	0.71	2.97	6.23	11.56
	PM10	44.36	28.96	17.00	22.34	33.48	55.22	142.44
	NO_2_	7.32	3.21	3.30	4.71	6.35	9.35	17.56
	O_3_	22.13	9.43	10.36	14.92	18.45	28.54	45.53
Northeastern	Incidence rate	1.12	1.37	0.10	0.29	0.53	1.31	7.43
	Temperature	27.21	2.15	20.62	25.89	27.63	28.56	32.24
	Relative Humidity	71.96	7.63	54.71	66.37	71.64	78.88	85.09
	Precipitation	4.03	3.41	0.05	1.00	3.12	6.72	13.00
	PM10	44.13	18.91	18.00	28.63	39.85	55.80	99.67
	NO_2_	15.45	6.23	6.00	10.88	14.00	19.13	33.00
	O_3_	22.34	8.84	8.00	16.00	20.25	26.50	47.00
Central	Incidence rate	1.39	1.43	0.09	0.47	0.79	1.78	7.29
	Temperature	27.79	1.73	22.08	26.79	27.82	28.79	32.15
	Relative Humidity	73.26	6.65	58.46	67.86	73.95	79.23	83.76
	Precipitation	3.46	2.94	0.00	0.69	3.01	5.73	12.12
	PM10	55.50	20.54	26.00	36.33	53.47	70.03	110.60
	NO_2_	13.68	4.46	7.50	10.00	13.00	16.15	26.60
	O_3_	24.96	7.70	11.33	17.80	24.45	31.45	42.00
Bangkok-Metropolitan	Incidence rate	3.27	3.57	0.11	0.91	1.90	4.23	16.64
	Temperature	28.89	1.38	24.46	28.28	28.95	29.69	31.75
	Relative Humidity	70.97	4.92	58.72	67.62	70.97	75.08	78.63
	Precipitation	3.89	3.31	0.00	0.87	3.28	6.37	13.34
	PM10	44.19	13.27	25.28	33.73	41.15	52.96	94.42
	NO_2_	20.20	6.42	10.20	15.45	18.05	25.04	36.86
	O_3_	18.47	5.82	9.41	13.32	18.53	23.19	33.05
Eastern	Incidence rate	1.89	2.09	0.18	0.55	1.02	2.53	11.71
	Temperature	28.22	1.14	24.33	27.58	28.21	28.87	30.93
	Relative Humidity	75.71	5.54	60.29	72.71	75.96	80.31	84.76
	Precipitation	5.33	4.26	0.00	1.54	4.78	8.40	16.76
	PM10	36.01	12.79	18.91	25.96	32.86	43.16	77.30
	NO_2_	10.54	2.77	5.90	8.39	10.29	12.03	19.00
	O_3_	21.76	7.20	9.70	15.86	20.58	27.12	44.56
Southern	Incidence rate	1.14	1.05	0.20	0.49	0.76	1.36	6.57
	Temperature	27.83	0.86	25.63	27.25	27.84	28.44	30.46
	Relative Humidity	78.42	3.37	70.55	76.43	78.71	80.85	84.67
	Precipitation	5.76	3.35	0.13	3.55	5.94	7.31	16.69
	PM10	30.94	6.14	18.50	26.40	30.00	34.81	56.40
	NO_2_	6.70	1.72	3.75	5.33	6.33	7.67	14.00
	O_3_	18.99	6.57	8.67	14.13	17.55	21.95	45.67

For meteorological variables, the average monthly temperature across all regions was 27.78 °C, ranging from a minimum of 20.51 °C in the N region to a maximum of 32.24 °C in the NE region. The average monthly relative humidity was 73.70 % for all regions, ranging from a minimum of 49.65 % in the N region to a maximum of 85.09 % in the NE region. For precipitation, the average value for all regions was 4.35 mm, varying from a minimum of 0 to a maximum of 16.76 mm in the E region. The details for each region are shown in Table 1. The N region exhibited the lowest temperature, relative humidity, and precipitation, while the NE region showed high temperature and relative humidity. The E and S regions had the highest precipitation due to their coastal locations, as illustrated on the map in Fig. 1. For air pollution, the average monthly concentrations of PM10, NO_2_, and O_3_ ranged from 17 to 142 μg/cm^3^, 3.3–36.9 ppb, and 8–47 ppb, respectively. The N region recorded the highest PM10 concentration, followed by the BKK region. The highest NO_2_ and O_3_ concentrations were found in the BKK and NE regions, respectively.

The time series data of influenza incidence rates displayed seasonality across all six regions (Figs. S1–S6). Most influenza incidence peaks correspond to the seasonality of meteorological factors and air pollutants. We found an annual cycle for temperature, relative humidity (RH), and precipitation. Moreover, the concentrations of PM10, NO_2_, and O_3_ exhibited seasonality with an annual peak during the winter season (from December to February). To investigate the relationships of all seven variables in each region, Spearman's correlation was calculated (Fig. S7). We found a strong positive correlation between RH and precipitation in all regions. PM10 concentration also correlated positively with the NO_2_ and O_3_ concentrations in five regions, with the exception of the S region, where PM10 concentration was only correlated with O_3_ concentrations and not with NO_2_ concentrations.

### 3.2 Continuous wavelet transforms of influenza incidence

**Fig. 2:**
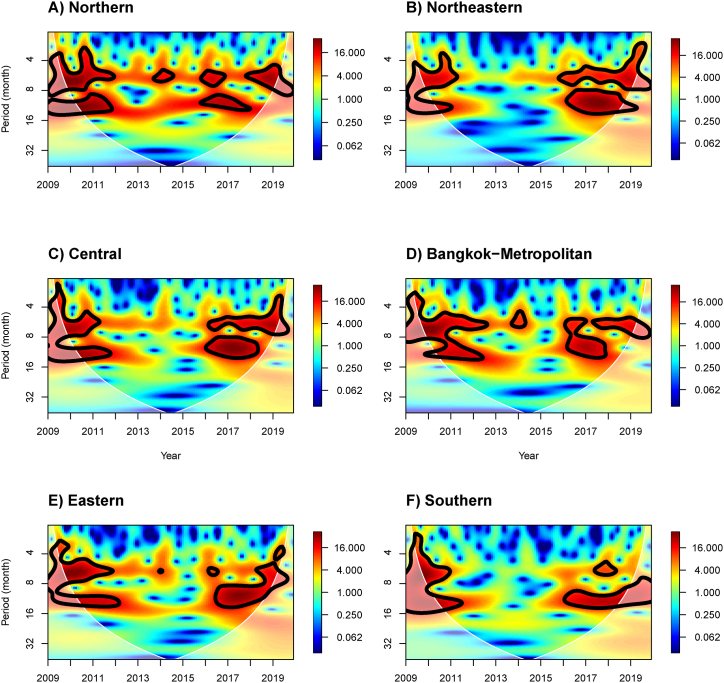
CWT spectra of influenza incidence for different six regions of Thailand: northern, northeastern, central, Bangkok-metropolitan, eastern, and southern from 2009 to 2019.

Fig. 2 demonstrates the continuous wavelet transform (CWT) of the influenza incidence for the six regions. The red area within the black contour indicates the significant periods at that time. The rainbow-colored bar (ranging from red to blue) shows the wavelet power spectrum from the highest to lowest. The cone of influence (COI) represents the edge effect, separated by the white cone-shaped area.

Overall, the CWT result of the influenza incidence exhibited two significant regions in the frequency-time domain during 2010–2012 with higher powers in the 2–8 and 10–14 month bands (Fig. 2). This result demonstrated the biannual influenza prevalence for all regions of Thailand during 2010–2012. It is remarkable that the periodicity disappeared, corresponding with the decrease in influenza incidence during 2013–2016 (Figs. S1–S6), except in the N, BKK, and E regions, where there was an island of high power in the 4–6 month band in 2014.

In the year 2016 and beyond, the rise in influenza incidence was consistent with the influenza periodicity that exhibited two areas of high power in the 4–8 and 8–14 month bands for both NE and C regions (Fig. 2B and C), corresponding with the biannual cycle of influenza incidence (Figs. S2–S3). For the N region (Fig. 2A), the CWT results showed two areas of high power in the 4–8 month band in 2016 and 2018–2019, while another area of high power in the 8–16 month band was observed during 2016–2018. For the BKK region (Fig. 2D), the CWT results showed two significant areas of high power: one in 2016–2018 with high power in the 6–16 month band, and another in 2017–2019 with high power in the 6–8 month band. For the E and S regions (Fig. 2E and F), there was one large area with high power in the 8–16 month band in 2016–2019 and a few islands of high power with a 4–6 month band.

We also applied the CWT of the meteorological and air pollution factors to demonstrate their periodicity (Figs. S8–S13). For the N, NE, C, BKK, and E regions, all variables exhibited a significant area with high power in the 10–14 month band during all studied periods, except for the O_3_ concentration in the NE region, which showed a high power area with a 2–8 month band in 2015. The CWT of temperature for the N, NE, C, BKK, and E regions demonstrated one more area of high power with a 4–8 month band during 2012–2017. For the S region, temperature exhibited periodicity with a 10–14 month band of period, but there was no periodicity observed for RH and precipitation in 2011–2012. For the concentrations of PM10, NO_2_, and O_3_, there were variations in periodicities in the S region.

### 3.3 Wavelet transform coherence

**Fig. 3:**
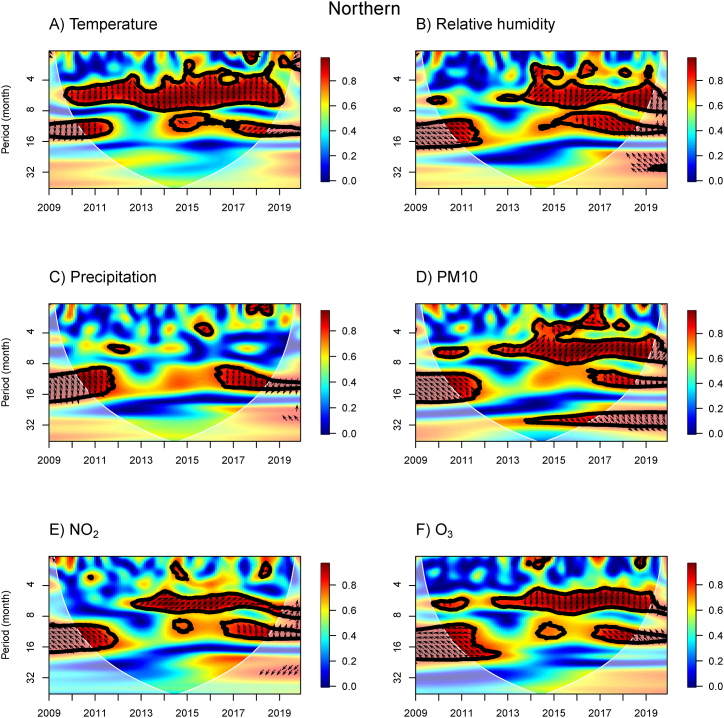
WTC of the influenza incidence with temperature, % relative humidity, precipitation, PM10, NO_2_ concentration, and O_3_ concentration in the Northern region of Thailand. A colored contour shows the wavelet transform coherence, which the red (blue) color is represented the high (low) correlation in the time-period domain (with time on the x-axis and period on the y-axis). The scales matching with the color are on the right-hand side of the graph. A thick black curve shows the 5 % significant level against red noise. The lighter shade area represents the cone of influence (COI) indicating the area affected by edge effects. Arrows show the phase difference between the two time-series. Arrows pointing to the right (left) mean that the variables are in phase (out of phase) or the variables have positive (negative) association. Arrows pointing down show that the meteorological or air pollution factors leads. Arrows pointing up mean that influenza prevalence leads. (For interpretation of the references to color in this figure legend, the reader is referred to the Web version of this article.)

**Fig. 4:**
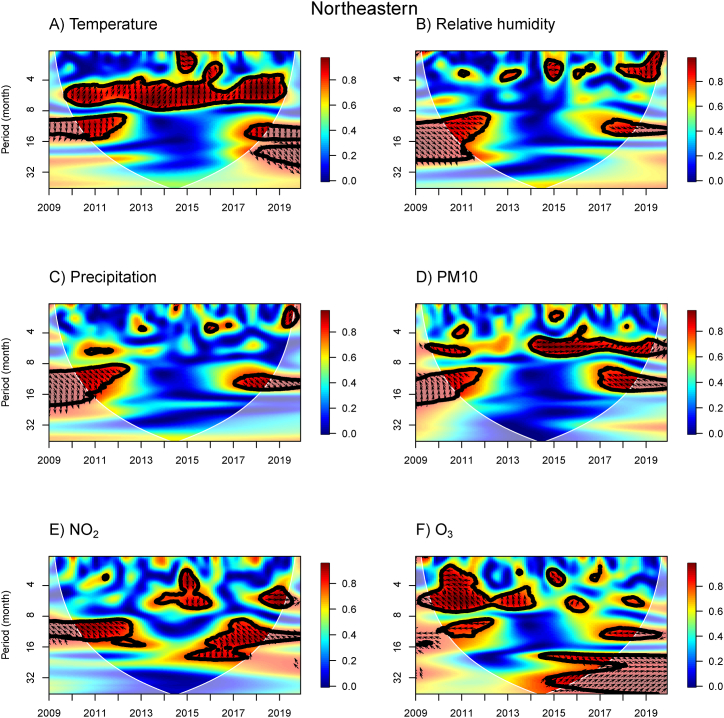
WTC of the influenza incidence with temperature, % relative humidity, precipitation, PM10, NO_2_ concentration, and O_3_ concentration in the Northeastern region of Thailand. A colored contour shows the wavelet transform coherence, which the red (blue) color is represented the high (low) correlation in the time-period domain (with time on the x-axis and period on the y-axis). The scales matching with the color are on the right-hand side of the graph. A thick black curve shows the 5 % significant level against red noise. The lighter shade area represents the cone of influence (COI) indicating the area affected by edge effects. Arrows show the phase difference between the two time-series. Arrows pointing to the right (left) mean that the variables are in phase (out of phase) or the variables have positive (negative) association. Arrows pointing down show that the meteorological or air pollution factors leads. Arrows pointing up mean that influenza prevalence leads. (For interpretation of the references to color in this figure legend, the reader is referred to the Web version of this article.)

**Fig. 5:**
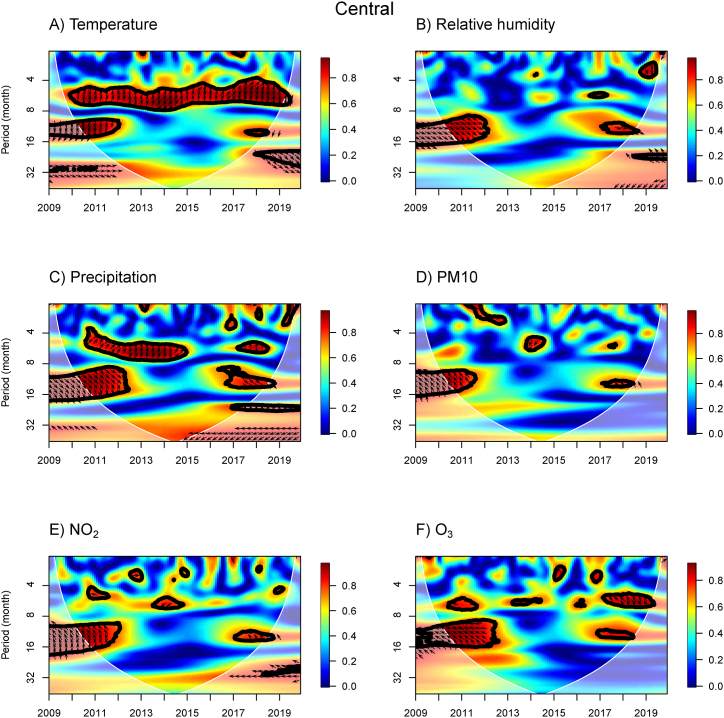
WTC of the influenza incidence with temperature, % relative humidity, precipitation, PM10, NO_2_ concentration, and O_3_ concentration in the central region of Thailand. A colored contour shows the wavelet transform coherence, which the red (blue) color is represented the high (low) correlation in the time-period domain (with time on the x-axis and period on the y-axis). The scales matching with the color are on the right-hand side of the graph. A thick black curve shows the 5 % significant level against red noise. The lighter shade area represents the cone of influence (COI) indicating the area affected by edge effects. Arrows show the phase difference between the two time-series. Arrows pointing to the right (left) mean that the variables are in phase (out of phase) or the variables have positive (negative) association. Arrows pointing down show that the meteorological or air pollution factors leads. Arrows pointing up mean that influenza prevalence leads. (For interpretation of the references to color in this figure legend, the reader is referred to the Web version of this article.)

**Fig. 6:**
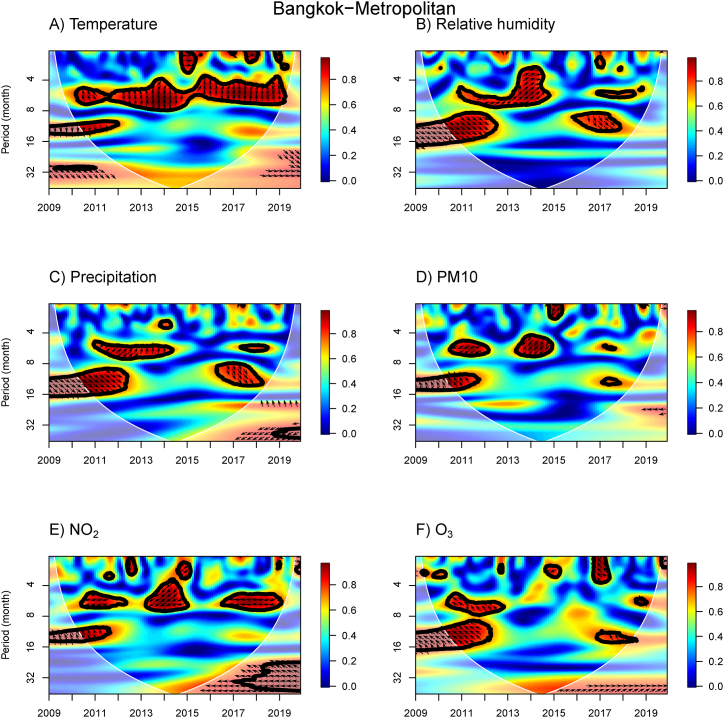
WTC of the influenza incidence with temperature, % relative humidity, precipitation, PM10, NO_2_ concentration, and O_3_ concentration in the Bangkok-Metropolitan. A colored contour shows the wavelet transform coherence, which the red (blue) color is represented the high (low) correlation in the time-period domain (with time on the x-axis and period on the y-axis). The scales matching with the color are on the right-hand side of the graph. A thick black curve shows the 5 % significant level against red noise. The lighter shade area represents the cone of influence (COI) indicating the area affected by edge effects. Arrows show the phase difference between the two time-series. Arrows pointing to the right (left) mean that the variables are in phase (out of phase) or the variables have positive (negative) association. Arrows pointing down show that the meteorological or air pollution factors leads. Arrows pointing up mean that influenza prevalence leads. (For interpretation of the references to color in this figure legend, the reader is referred to the Web version of this article.)

**Fig. 7:**
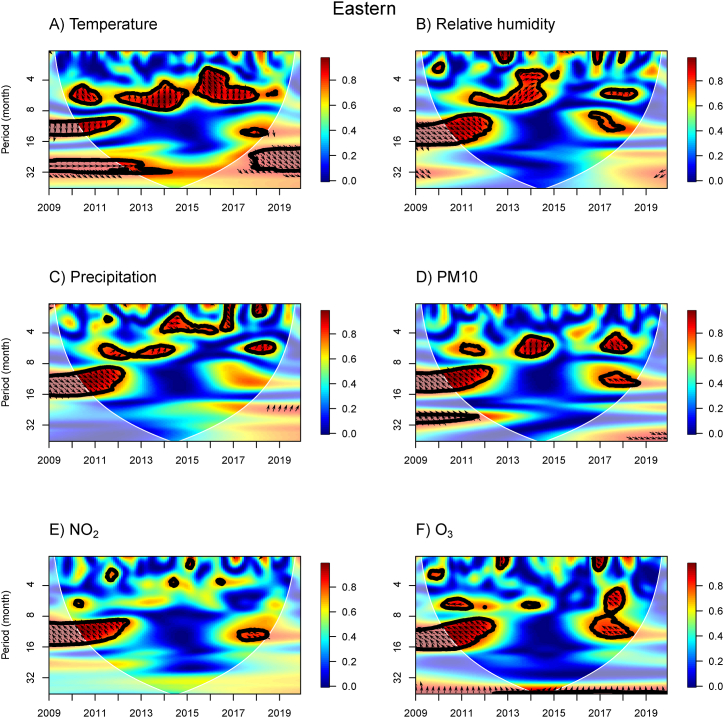
WTC of the influenza incidence with temperature, % relative humidity, precipitation, PM10, NO_2_ concentration, and O_3_ concentration in the Eastern region of Thailand. A colored contour shows the wavelet transform coherence, which the red (blue) color is represented the high (low) correlation in the time-period domain (with time on the x-axis and period on the y-axis). The scales matching with the color are on the right-hand side of the graph. A thick black curve shows the 5 % significant level against red noise. The lighter shade area represents the cone of influence (COI) indicating the area affected by edge effects. Arrows show the phase difference between the two time-series. Arrows pointing to the right (left) mean that the variables are in phase (out of phase) or the variables have positive (negative) association. Arrows pointing down show that the meteorological or air pollution factors leads. Arrows pointing up mean that influenza prevalence leads. (For interpretation of the references to color in this figure legend, the reader is referred to the Web version of this article.)

**Fig. 8:**
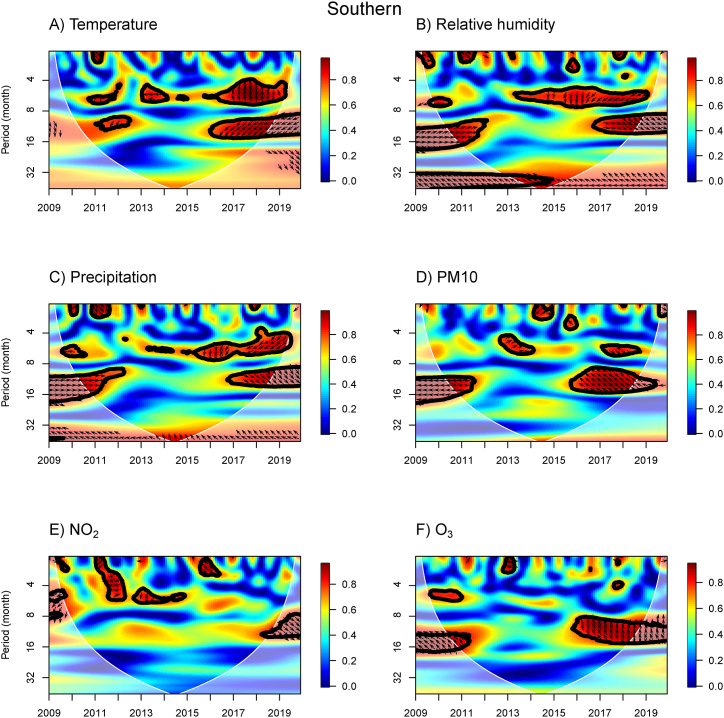
WTC of the influenza incidence with temperature, % relative humidity, precipitation, PM10, NO_2_ concentration, and O_3_ concentration in the Southern region of Thailand. A colored contour shows the wavelet transform coherence, which the red (blue) color is represented the high (low) correlation in the time-period domain (with time on the x-axis and period on the y-axis). The scales matching with the color are on the right-hand side of the graph. A thick black curve shows the 5 % significant level against red noise. The lighter shade area represents the cone of influence (COI) indicating the area affected by edge effects. Arrows show the phase difference between the two time-series. Arrows pointing to the right (left) mean that the variables are in phase (out of phase) or the variables have positive (negative) association. Arrows pointing down show that the meteorological or air pollution factors leads. Arrows pointing up mean that influenza prevalence leads. (For interpretation of the references to color in this figure legend, the reader is referred to the Web version of this article.)

The WTC results provide information about the association between influenza incidence and meteorological and air pollution variables for the N, NE, C, BKK, E, and S regions (Fig. 3, Fig. 4, Fig. 5, Fig. 6, Fig. 7, Fig. 8). The power spectra within the contour indicate significant associations, with the color bar on the right illustrating their strength. Arrows pointing to the right (left) indicate a positive (negative) association between the two variables. Downward arrows indicate that the meteorological factors, or air pollutant concentrations lead the influenza prevalence by 90° (negative phase difference), indicating potential driving factors for influenza incidence in specific times and periods. To fully understand and interpret the direction (lead/lag) of the relationship between influenza incidence and exposure variables, we summarize the arrows from the WTC spectra (Fig. 3, Fig. 4, Fig. 5, Fig. 6, Fig. 7, Fig. 8) as the average phase differences at periods of 6 and 12 months, shown in Figs. S14 and S15, respectively. The phase difference is measured in degrees, with a full cycle of 360° corresponding to a specific period. For a period of 6 (12) months, a phase difference of 90° indicates that influenza incidence leads the exposure variables by a quarter of a period, which is 1.5 (3) months.

In the N region, the WTC results revealed associations between influenza incidence and various weather and air pollution factors during specific periods (Fig. 3). The WTC results for temperature showed a significant association around a 10–14 month band in 2010–2012 and 2017–2018, with downward arrows indicating that temperature leads incidence by approximately 3 months in 2010–2012 and 5 months in 2017–2018. In 2014, the arrows pointing up-left suggested that the incidence of influenza leads the temperature. Similarly, the power spectrum around a 4–8 month band showed that influenza incidence leads temperature (the pointing up arrows). For RH, there were two large areas of association at the period of 10–18 month bands in 2010–2011 and 2015–2018, as well as another association at a 3–7 month band in 2013–2019. We can interpret that RH leads the incidence by 3–4 months for the 3–7 month band and 1–2 months for the period of 10–18 month band (arrows pointing down). For precipitation, the WTC results also showed associations at the 9–18 month band in 2010–2012 and 2016–2019, with an island of high power observed in 2015 at approximately 4 months of the period in 2016. There were similar results for relative humidity and precipitation because they have a strongly positive correlation (see Fig. S7). For the concentrations of PM10, NO_2_, and O_3_, we observed some associations at the 4–8 and 8–16 month bands with the arrows pointing up, showing that influenza incidence leads air pollutants. However, for the period less than 4 months, we found an island in 2016 for PM10 and in 2018 for NO_2_ concentration have a negative association with the incidence (arrows pointing to the right).

In the NE region, the WTC analysis of influenza with temperature, RH, and precipitation (Fig. 4A–C) showed similar results to the N region. Additionally, a significant association was observed between influenza incidence and NO_2_ concentrations at periods of approximately 3–6 month band in 2015, with downward-pointing arrows indicating that the NO_2_ concentration leads by around 3 months (Fig. 4E). High power was also present in a 4–6 month band in 2018–2019, with right-pointing arrows suggesting a positive association between NO_2_ concentration and influenza incidence or occurring at the same time. For the O_3_ concentration, negative associations were observed at periods of 9–12 month band in 2010–2012 and 2017–2019, while positive associations were observed at a 16–18 month band in 2014–2019, indicating that O_3_ concentration leads influenza prevalence by approximately 4 months.

In the C and BKK regions (Fig. 5, Fig. 6B), we also observed the WTC power spectra for the associations of influenza incidence with temperature and RH, consistent with the results in the N and NE regions for the years 2010–2012. However, associations with temperature disappear around the 8–16 month band in 2017 and 2017–2019 in the C, and BKK regions (Fig. 6A), respectively. The significant area for the association between RH and influenza was also smaller around the 8–16 month band in the year 2017–2019 for these two regions. For precipitation, a positive association at the 4–8 month bands in 2011–2014 and 2018 (Fig. 5, Fig. 6C) was indicated by arrows pointing up and up-right, suggesting that precipitation lags by approximately 1–3 months. These results contrast with the association of RH in the C region, despite the strong positive correlation between RH and precipitation (Fig. S7). For the PM10 concentration in the C region (Fig. 5D), we found a negative association between PM10 concentration and influenza incidence over around 4–5 month band in 2014, with arrows pointing down-left indicating that PM10 concentration leads by approximately 4 months. In the BKK region (Fig. 6D), we found two negative associations of PM10 concentration in a 4–6 month band in 2011–2012 and 2014, suggesting that PM10 concentration leads by approximately 4–5 months. Similar to PM10 concentration, there was an association of NO_2_ concentration over around 4–6 month band in 2014, but NO_2_ concentration leads by 4–5 months in both C and BKK regions (Fig. 5, Fig. 6E). That aligns well with the positive correlation results between PM10 and NO_2_ concentrations. For the O_3_ concentration, we observed negative associations with various periods in different years (Fig. 5, Fig. 6F).

In the E regions (Fig. 7), we observed the associations between temperature and influenza incidence over a 10–13 month band in 2010–2012 and a small area of high power in 2017–2018. In these areas, the arrows pointing down by 90° indicate the temperature leads by 3 months. We also found a positive association of RH and precipitation with influenza incidence at the period of 9–17 month band in 2009–2012 with the arrows pointing right downward, which suggests that the RH and precipitation lead by 1 month. We also found a positive association between the PM10 concentration and influenza incidence over the period of 4–6 month bands in 2013–2014 (PM10 leads 2 months) and 2017–2018 (PM10 leads 1 month).

In the S region (Fig. 8), we found a negative association of temperature over a period of 10–15 month band only in 2016–2019, with temperature leading by 4 months. We did not find the same association in 2010–2012 as we found in other regions. For RH and precipitation, we found negative associations with the 5–6 month band in 2013–2019, indicating that RH and precipitation lead by 3–5 months. At the period of 9–12 month band, we identified two areas of positive association in 2010–2012 and 2017–2019, where RH and precipitation nearly coincide with influenza incidence. Regarding air pollution, we found small areas of associations over a period of 4–6 month band in 2017–2018 for PM10 concentration and in 2010 for O_3_ concentrations, indicating that air pollution leads.

### 3.4 Partial wavelet coherence (PWC) and multiple wavelet coherence (MWC)

Our WTC results show that both meteorological factors and air pollution correlated with influenza incidence over specific time-period domain. To study the effect of each variable, we examined two groups of PWC, representing the partial effects of meteorological factors (Fig. S16) and air pollution (Fig. S17).

For the three meteorological variables, we first applied the PWC for temperature by excluding RH (TEMP|RH) and precipitation (TEMP|PRCP) to observe the effect of temperature independently (Fig. S16). Most of the high PWC for the impact of temperature by excluding RH and precipitation were found over approximately a 4–8 month band in 2013–2018 in the N, NE, C, and BKK regions, except the E and S regions, which had many small islands. However, the WTC results for the same time-period domain showed that temperature has a negative association with influenza incidence and lags influenza incidence (Fig. 3, Fig. 4, Fig. 5, Fig. 6, Fig. 7, Fig. 8). For the 8–16 month band, there was high WTC and low PWC, suggesting that temperature did not affect influenza incidence across all regions. Therefore, we concluded that temperature did not have a significant impact on influenza incidence in those time-period domains.

We also focused on RH and precipitation without the effect of temperature (RH|TEMP and PRCP|TEMP). It is important to note that RH and precipitation are highly correlated (Fig. S7). Thus, we do not consider them as independent variables for PWC analysis. For RH|TEMP, we found a high PWC of around an 8–16 month band in 2016–2019 across all regions, except the S region. This area corresponds with the high WTC between RH and influenza incidence, implying that RH leads influenza incidence. In addition, a high PWC around this month band was also found in 2010–2012 for the BKK and E regions. Therefore, during these time-period domains, RH has an impact on influenza incidence across all regions of Thailand, except the S region. For PRCP|TEMP, high PWC was found around the 8–16 month band in 2016–2018 for the NE, C, BKK, and E regions, corresponding with high WTC. We can infer that precipitation can influence the influenza incidence in the NE, C, BKK, and E regions, but not in the N and S regions. Across almost all regions of Thailand (excluding the S region), RH can be the main driver for the influenza incidence around the 8–16 month band in 2010–2012 and 2016–2018, whereas precipitation did not show the effect for N and S regions.

We also investigated the PWC of influenza incidence and the concentrations of air pollution (Fig. S17). We observed the influence of PM10 concentration without the effect of NO_2_ and O_3_ concentrations (PM10|NO_2_ and PM10|O_3_). The association in the 4–8 month band disappears for the effect of NO_2_ and O_3_ concentrations when removing the impact of PM10 concentrations (NO_2_|PM10 and O_3_|PM10), showing that PM10 concentration was responsible for the influenza incidence. Additionally, PM10 concentration was responsible for the influenza prevalence around the period of 4–8 month band in 2014 for the C, BKK, and E regions.

Therefore, we combined the impact of RH and PM10 concentration on influenza incidence using MWC analysis in Fig. S18. The linear combination of RH and PM10 concentration can explain the co-movement between incidence and these two factors in almost all periods, including the 0–4, 4–8, and 8–16 month bands during 2009–2019. Based on MWC results, we can confirm that the combination of RH and PM10 concentration were the main factors influencing influenza incidence in Thailand.

## 4 Discussion

In this study, we investigated influenza seasonality and the impact of exposure variables in Thailand from 2009 to 2019 using wavelet analysis and wavelet coherence. The continuous wavelet transform (CWT) analysis reveals a clear pattern in influenza incidences, indicating a distinct periodic mode. We found that the CWT exhibited two significant regions in the frequency-time domain during 2010–2012 and 2016–2019, with higher powers across all regions. Our results highlight the biannual influenza epidemic only during 2010–2012. A previous study conducted in Thailand from 2004 to 2010 found that the occurrence of influenza cases had two seasonal waves in the rainy and winter seasons [41], which supports our findings for the period before 2012 across all regions.

Interestingly, we observed no periodic pattern from 2013 to 2016 except in the northern and Bangkok metropolitan regions. We found a distinct epidemic peak with high power over a 6-month period in 2014. Various periodic patterns were identified in Amapá, Brazil, near the equator line, where no consistent pattern was found [42]. However, Paraná, located in the South, exhibited a seasonal signal over a 52-week period from 2010 to 2016 [42]. Unexpectedly, our findings contradict those of studies conducted in Bangkok using data from 2010 to 2018 [11] and in Chiang Mai from 2010 to 2020 [25], which identified a biannual pattern. It is important to note that these studies were limited to only one province and may not represent the seasonal pattern of the entire region. However, the disappearance of periodicity during 2013–2016 needs further investigation. Other climate factors, such as the ENSO index might be responsible for the no periodicity. A study in New York State suggested that a low ENSO index may drive influenza prevalence with 2-month lag [43]. Wavelet analysis allows us to investigate the non-normal and non-stationary time series by localizing on specific interactions within the time-frequency domain [44]. Our results highlight the distinct periodic patterns associated with specific frequencies and time rather than the overall time series. The seasonal pattern of influenza in each region may relate to the seasonality of climate factors or air pollution. Therefore, we explored the relationships between influenza incidence and several factors through WTC analysis.

Overall, we found that high relative humidity and precipitation lead the incidence of influenza ranging from a 9–18 month band in during 2010–2012 and 2016–2019. However, in the eastern and southern regions of Thailand, we found different influences of relative humidity and precipitation compared to other regions (Figs. S14 and S15). The Eastern and Southern regions of Thailand are coastal areas adjacent to the sea, and their climate is influenced by the Gulf of Thailand and the Pacific Ocean, which may account for the different results. Notably, this is the first report for the eastern region, revealing that relative humidity and precipitation influenced the influenza incidence over a 12-month period only in 2010–2012. Relative humidity also has an effect in 2017–2018, while precipitation does not. In the southern region, we found that the relative humidity and precipitation nearly coincided with influenza incidence in 2010–2011. Relative humidity and precipitation also drive the influenza rate at a period of 6 months in 2014–2019 at which these results cannot be seen in other regions. In the central and Bangkok-metropolitan regions, our finding for both climate factors agree with a previous study in Bangkok [11], but not with another study [24], which found no correlation between rainfall and influenza cases in central Thailand. A study in Chiang Mai, Northern Thailand, found no association between influenza incidence and relative humidity [25]. Increased influenza activity in Bangkok was associated with increased relative humidity using time series analysis [11]. In contrast, studies in temperate zone have observed a negative association between relative humidity and influenza [13,36]. However, a study in Toronto, Ontario, Canada, found an increased trend of influenza A with relative humidity [45]. For rainfall, a positive correlation has been suggested in Guatemala's central departments and Panama province, in Brazil [16], French Guiana [17], and Spain [46]. However, the finding of the association of relative humidity and rainfall has been suggested to vary across latitudes [13].

For a one-year period band in the northern (Fig. 3A), central (Fig. 5A) and southern (Fig. 8A) regions, temperature has a trend to be a negative correlation with influenza incidence. Increased influenza incidence relates to low temperatures. The association between low temperature and influenza was found in temperate regions, such as the study in New York State [43] and Jinan City, Eastern China [36]. In all regions, low temperatures are observed in December–January (mid-winter season) before the influenza peaks during the winter season, whereas high temperature in the summer season are observed prior to influenza peaks during the rainy season. Our study demonstrates that the effect of temperature can differ across regions. This may be due to the range of temperature having different effects. A study in the Fujian province of China found that high temperature (>23 °C) was one of the risk factors for influenza [47]. The maximum relative risk of influenza-like illness was found to be caused by high temperature (26 °C) on the same day in Jiangsu Province, China [48]. At 30 °C, influenza transmission was blocked or inefficient using the guinea pig model [49].

Our study provides a novel contribution to understanding the impact of air pollution, revealing remarkably different effects on influenza incidence across six regions of Thailand. For a 4–8 month band, we found similar effects in central, Bangkok-metropolitan, eastern, and southern parts in some years. PM10, particle matters smaller than 10 μm generated from various sources such as the combustion in the field or industrial sites, and grinding at the construction sites [31], can be vectors or carriers for the virus, facilitating airborne transmission [50]. Infected individuals exhale the virus, and PMs spread it in the air. Thus, high PM10 concentrations increase virus transmission via aerosols and droplets [50]. According to Murtas's study, there is a statistically significant association between PM10 and influenza for cardiovascular-related mortality, showing that high levels of PM10 (60–70 μg/m^3^) increase mortality from respiratory diseases [51]. A study in Bialystok, Poland, also showed an association between influenza-like illness (ILI), meteorological variables, and PM concentrations from 2013 to 2019. They found that high PM concentrations in the winter season were associated with high ILI and other respiratory infections [52]. Moreover, a study in China discovered that the increasing of air pollutants such as PM2.5, PM10, and other chemicals, were positively associated with ILI at different lags, especially in the cold season and in the eastern and central regions of China [53]. A study in Warsaw also found similar results, indicating that high PM2.5 and PM10 concentrations increased ILI morbidity in cold atmospheric conditions [19]. Interestingly, the low PM10 concentrations were found to correlate with influenza in central and Bangkok-metropolitan areas in 2014–2015. A similar effect was found in studies conducted in Hefei, China [54], and Singapore [55]. Some previous studies suggested that the increased PM10 concentrations may result in less exposure to ultraviolet radiation, potentially contributing to influenza transmission [[54], [55], [56]]. However, these factors are not clear and need further investigation.

For NO_2_ concentration, we found a positive association with influenza only in the northeastern region. Similar to the effect of PM10 concentration, we found an association in central and Bangkok-metropolitan due to the high correlation with PM10. Our results indicated that NO_2_ concentration may not be the main seasonal driver in Thailand. Exposure to NO_2_ may affect in the immune system leading to a higher incidence of influenza infection [57,58]. However, some previous studies found no significant relationship between NO_2_ and influenza [59,60].

Our results clearly illustrate that O_3_ concentration has a negative correlation with influenza incidence across all regions, but only in some years. This finding is supported by previous work in China [21,61] and in Hong Kong [62]. Ozone may have the ability to control and kill bacteria depending on its type and application [63,64]. The effect of ozone can vary depending on the concentration, and only certain concentrations could reduce the infection [61,65]. Hence, this factor requires to be further investigation.

PWC results indicated that relative humidity could be the primary influencing factor for influenza incidence for a one-year period across all regions except the southern region. Precipitation has a similar influence on northeastern, central, Bangkok-metropolitan, and eastern regions. Both relative humidity and precipitation have no impact on the southern region. A significant difference between the effects of relative humidity and precipitation was observed only in the northern region. These variations in results can be attributed to various factors, including geographic variation, varying climate patterns, and socio-economic conditions. Additionally, we also found that PM10 concentration influences influenza incidence over a half-year period in some regions. Consequently, we decided to combine the effect of relative humidity and PM10 concentration on the influenza incidence by using MWC. Overall, we found that these two factors can be the main drivers for the seasonality of influenza incidence in Thailand from 2010 to 2012 and 2016 to 2019. However, more extensive studies are needed to fully understand the impact of climate and air pollution on influenza incidence in the eastern and southern regions.

Our findings include several interesting discoveries. First, this is the first study to investigate the effects of both weather and air pollution factors across six regions in Thailand. Second, the study provides insights into the seasonal dynamics of influenza, aiding in the understanding of its occurrence. This knowledge can support vaccination campaigns, one of the main control measures. Third, relative humidity and PM10 concentration contribute to the seasonality of influenza in different periods and regions. Thus, controlling PM10 concentrations could help reduce influenza transmission.

## 5 Limitations

Our study has some limitations. First, we did not have information on influenza virus types and subtypes at the regional level, which can be related to the seasonality and impact of climate and air pollution factors. This might be responsible for the diverse seasonal patterns in each region. The importance of considering vaccination timing or other measurement in the event of a new virus pandemic should be a concern. Second, the reported data is derived mostly from public hospitals and some private hospitals. The data may underreport influenza cases, as some infected individuals with influenza with mild symptoms may not go to the hospital and may stay at home. However, the underreported cases tend to be random and have no impact on seasonal patterns. Hence, our study can show the actual burden of influenza incidence in the regions. Moreover, we only considered meteorological factors such as temperature, relative humidity, and precipitation due to data availability. Other factors like absolute humidity were not included, even though they may affect the prevalence of influenza incidence. Finally, our analysis did not incorporate continuous monitoring of other air pollutants such as PM2.5, CO, or SO_2_ concentrations. The available air pollution data for the long-term period is provided on a monthly scale. Future studies at finer time scales, such as weekly or daily, should be conducted when available to better understand the impact on influenza incidence. These pollutants could potentially contribute to the observed seasonal influenza patterns [20]. Further research should aim to include a broader range of contributing factors to better understand the complex dynamics of influenza incidence.

## 6 Conclusion

Wavelet transform analysis demonstrates the periodicity of influenza incidence and provides insight into the impact of meteorological factors and air pollution on influenza incidence in Thailand during 2009–2019. Our findings reveal inconsistent biannual incidence patterns throughout the study period. The biannual pattern was found in all regions from 2010 to 2012 but vanished from 2013 to 2016. However, after 2016, the biannual cycles reappeared, with peaks typically occurring during the rainy and winter seasons in most regions, except for the southern region. Furthermore, wavelet coherence reveals that relative humidity can be the main influencing factor for influenza incidence over a one-year period in the northern, northeastern, central, Bangkok-metropolitan, and eastern regions, not in the southern region during 2010–2012 and 2016–2018. Similarly, precipitation can drive the influenza incidence at the same period for the northeastern, central, Bangkok-metropolitan, and eastern regions. PM10 concentration can influence influenza incidence over a half-year period in the northeastern, central, Bangkok-metropolitan, and eastern regions of Thailand during certain years. These findings substantially enhance our understanding of the temporal characteristics of influenza outbreaks, offering valuable insights for the construction of predictive models that can effectively inform public health strategies and decisions in pertinent contexts.

## Ethical standards

All information on influenza surveillance data was collected from the Division of Epidemiology, Department of Disease Control, the Ministry of Public Health, Thailand. This study was approved by the Institutional Review Board of Naresuan University (IRB No. P1-0075/2566) as Exemption Review.

## CRediT authorship contribution statement

**Suparinthon Anupong:** Writing – review & editing, Writing – original draft, Methodology, Investigation, Formal analysis. **Charin Modchang:** Writing – review & editing, Validation, Resources. **Sudarat Chadsuthi:** Writing – review & editing, Writing – original draft, Validation, Supervision, Project administration, Investigation, Funding acquisition, Formal analysis, Conceptualization.

## Declaration of competing interest

The authors declare that they have no known competing financial interests or personal relationships that could have appeared to influence the work reported in this paper.
