# Supplementary material for: Seasonal patterns of influenza incidence and the influence of meteorological and air pollution factors in Thailand during 2009–2019
Source: Heliyon. 2024 Aug 22;10(17):e36703. doi: 10.1016/j.heliyon.2024.e36703 (PMC11388739; doi:10.1016/j.heliyon.2024.e36703)
Supplement: Multimedia component 1 [file mmc1.docx]

**Supplementary information**

**Seasonal Patterns of Influenza Incidence and the Influence of Meteorological and Air Pollution Factors in Thailand during 2009-2019**

Suparinthon Anupong^a^, Charin Modchang^b,c,d^ and Sudarat Chadsuthi^e*^

^a^Department of Chemistry, Mahidol Wittayanusorn School (MWIT), Salaya, Nakhon Pathom 73170, Thailand

^b^Biophysics Group, Department of Physics, Faculty of Science, Mahidol University, Bangkok 10400, Thailand

^c^Centre of Excellence in Mathematics, MHESI, Bangkok 10400, Thailand

^d^Thailand Center of Excellence in Physics, Ministry of Higher Education, Science, Research and Innovation, 328 Si Ayutthaya Road, Bangkok 10400, Thailand

^e^Department of Physics, Faculty of Science, Naresuan University, Phitsanulok 65000, Thailand

***Corresponding author:** Sudarat Chadsuthi

Department of Physics, Faculty of Science, Naresuan University, Phitsanulok 65000, Thailand

E-mail: sudaratc@nu.ac.th

**A: Wavelet transform analysis**

1) Continuous wavelet transforms (CWT)

CWT shows the time-frequency domain to analyze nonstationary time series, in which the frequency changes over time. The base function of CWT is a ‘Morlet mother wavelet’ as follows:

$\psi\left( t \right)= \pi^{-1/4}e^{i\omega\eta}e^{-\frac{\eta^{2}}{2}},$ (1)

where $\eta$ is dimensionless time $(\eta=1, \ldots, T)$, and $\omega$ is the dimensionless angular frequency, set to 6 radians per time [1]. The Morlet wavelet transform was applied as the bandpass filter to the time series by varying its scaled ($s)$ that is varied to stretch the wavelet in time. Therefore, $\eta=s\cdot t$ and the wavelet is normalized to have the unit energy. The CWT of a time series ($x_{1}, x_{2}, x_{3}, \ldots, x_{N})$ with the time step, $\delta t$, is

$W_{n}^{x}\left( s \right)=\sqrt{\frac{\delta t}{s}}\sum_{\acute{n}=1}^{N} x_{\acute{n}}\psi\left( \acute{n}-n \right)\frac{\delta t}{s},$ (2)

where $n=1,\ldots, N$ and $x_{\acute{n}}$is the input time series. The wavelet transform is calculated faster by using the convolution in Fourier space (see details in [2]). The conjugated complex of $W_{n}^{x}\left( s \right)$ can be indicated as the local phase. Wavelet power spectrum is defined as $\left| W_{n}^{x}\left( s \right) \right|^{2}$, which is shown in the time-period domain as the colored contour. The thick black curve shows the 5% significant level. The cone of influence (COI) indicates the area affected by edge effect, shown as the lighter area in power wavelet spectrum.

2) Wavelet transform coherence (WTC)

Wavelet coherence was utilized to investigate the connections between influenza incidence and meteorological factors, as well as between influenza incidence and air pollution. We applied WTC to search for the co-movement of two different time series, $X$ and $Y$. The WTC is defined as follows:

$R^{2}=\frac{\left| S\left( s^{-1}W_{n}^{XY}(s) \right) \right|^{2}}{S(s^{-1}\left| W_{n}^{X}(s) \right|^{2})\cdot S(s^{-1}\left| W_{n}^{Y}(s) \right|^{2})}$, (3)

where $W_{n}^{X}$and $W_{n}^{Y}$ are WTC of the time series $X$ and $Y$. The range of $R^{2}$ is between 0 and 1 which means no coherence for 0 and perfect coherence for 1. The Monte Carlo approach is used to determine significance levels by generating synthetic red noise data that is simulated from the observed data [1]. Wavelet transforms are then performed on both the synthetic and observed data to calculate wavelet power spectra. Significant regions in the observed data are identified by comparing these spectra. We can interpret the WTC as the covariance between two-time series. The contour lines indicate the consistency of their periodicities. We can capture bi-directional (lead-lag) relationships between different time-period combinations.

3) Partial wavelet coherence (PWC)

Several features (meteorological factors and air pollutants) might correlate with influenza incidence. To study the effect of each feature, we used the partial wavelet coherence (PWC) focusing on one feature effect with eliminating the others, which is a similar to the partial correlation [3]. The PWC is the result of WTC between two time series $Y$ and $X_{1}$ by removing the influence of the time series $X_{2}$. WTC between $Y$ and $X_{1}$, $Y$ and $X_{2}$, and $X_{1}$ and $X_{2}$ are shown as

$R\left( {Y,X}_{1} \right)= \frac{S\left[ W^{YX_{1}} \right]}{\sqrt{S\left[ W^{Y} \right]\cdot S\left[ W^{X_{1}} \right]}};$ (4)

$R^{2}\left( {Y,X}_{1} \right)=R\left( Y,X_{1} \right)\cdot{R\left( Y,X_{1} \right)}^{*};$ (5)

$R\left( {Y,X}_{2} \right)= \frac{S\left[ W^{YX_{2}} \right]}{\sqrt{S\left[ W^{Y} \right]\cdot S\left[ W^{X_{2}} \right]}};$ (6)

$R^{2}\left( {Y,X}_{2} \right)=R\left( Y,X_{2} \right)\cdot{R\left( Y,X_{2} \right)}^{*};$ (7)

$R\left( X_{2},X_{1} \right)= \frac{S\left[ W^{X_{2}X_{1}} \right]}{\sqrt{S\left[ W^{X_{2}} \right]\cdot S\left[ W^{X_{1}} \right]}};$ (8)

$R^{2}\left( X_{2},X_{1} \right)=R\left( X_{2},X_{1} \right)\cdot{R\left( X_{2},X_{1} \right)}^{*};$ (9)

$RP^{2}\left( Y,X_{1},X_{2} \right)=\frac{\left| R\left( {Y,X}_{1} \right)-R\left( {Y,X}_{2} \right)-{R\left( Y,X_{1} \right)}^{*} \right|^{2}}{\left[ 1-R\left( {Y,X}_{2} \right) \right]^{2}\left[ 1-R\left( X_{2},X_{1} \right) \right]^{2}}$. (10)

Whereas $R$ represents the coherence between two time series. WTC between all possible three covariances are calculated in eqs. (4 – 9). PWC squared (after the elimination of the effect of $X_{2}$) is determined by an equation similar to the partial correlation squared in eq. (10), which $RP^{2}$ is ranging from 0 to 1.

PWC squared ($RP^{2}\left( Y,X_{1},X_{2} \right)$) shows the correlation between $Y$ and $X_{1}$with the removal of $X_{2}$. In the period-time domain, a position that has a low value of PWC squared at where a high value of WTC squared of $Y$ and $X_{1}$was implied that the time series $X_{1}$ does not affect significantly to time series $Y$ and $X_{2}$ was consider as the dominant of the time series $Y$.

4) Multiple wavelet coherence (MWC)

Similar to PWC, multiple wavelet coherence (MWC) works like the multiple correlation, which gives the wavelet coherence of time series $Y$ depending on the linear combination of two independent time series $X_{1}$ and $X_{2}.$ MWC, $RM^{2},$ is defined as follows [3]:

$RM^{2}\left( Y,X_{1},X_{2} \right)=\frac{R^{2}\left( {Y,X}_{1} \right)+R^{2}\left( {Y,X}_{2} \right)-2Re\left[ R\left( {Y,X}_{1} \right)\cdot{R\left( {Y,X}_{2} \right)}^{*}\cdot{R\left( X_{2},X_{1} \right)}^{*} \right]}{1-R^{2}\left( X_{2},X_{1} \right)}$. (11)

In our study, the MWC result shows the effect of the combined two independent variables on the seasonal pattern of influenza incidence. The significant levels were calculated by using Monte Carlo method for both PWC and MWC.

1. Grinsted, A., Moore, J.C. and Jevrejeva, S., *Application of the cross wavelet transform and wavelet coherence to geophysical time series.* Nonlin. Processes Geophys., 2004. **11**(5/6): p. 561-566, 10.5194/npg-11-561-2004.

2. Torrence, C. and Compo, G.P., *A practical guide to wavelet analysis.* Bulletin of the American Meteorological society, 1998. **79**(1): p. 61-78.

3. Ng, E.K.W. and Chan, J.C.L., *Geophysical Applications of Partial Wavelet Coherence and Multiple Wavelet Coherence.* Journal of Atmospheric and Oceanic Technology, 2012. **29**(12): p. 1845-1853, <https://doi.org/10.1175/JTECH-D-12-00056.1>.

**B: Data and Results**

**
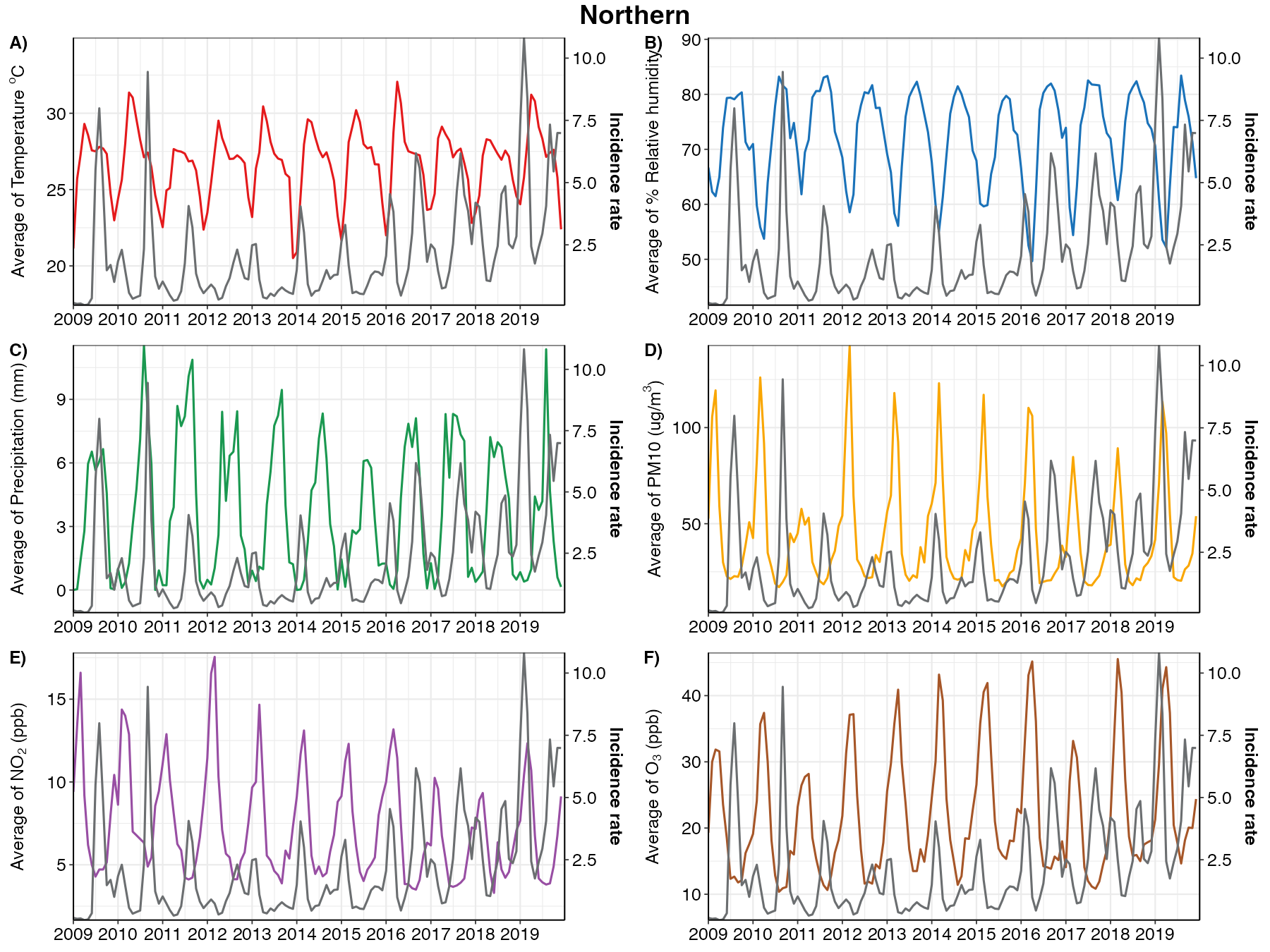
**

**Figure S1:** The time series of monthly influenza rate (cases per 10,000 population), showing in the grey color lines, combining with average temperature (^o^C) (red), average relative humidity (%) (blue), precipitation (mm) (green), PM10 (mg/m^3^) (yellow), NO_2_ (ppb) (purple), and O_3_ (ppb) (brown) in **Northern** region of Thailand during 2009-2019.

**
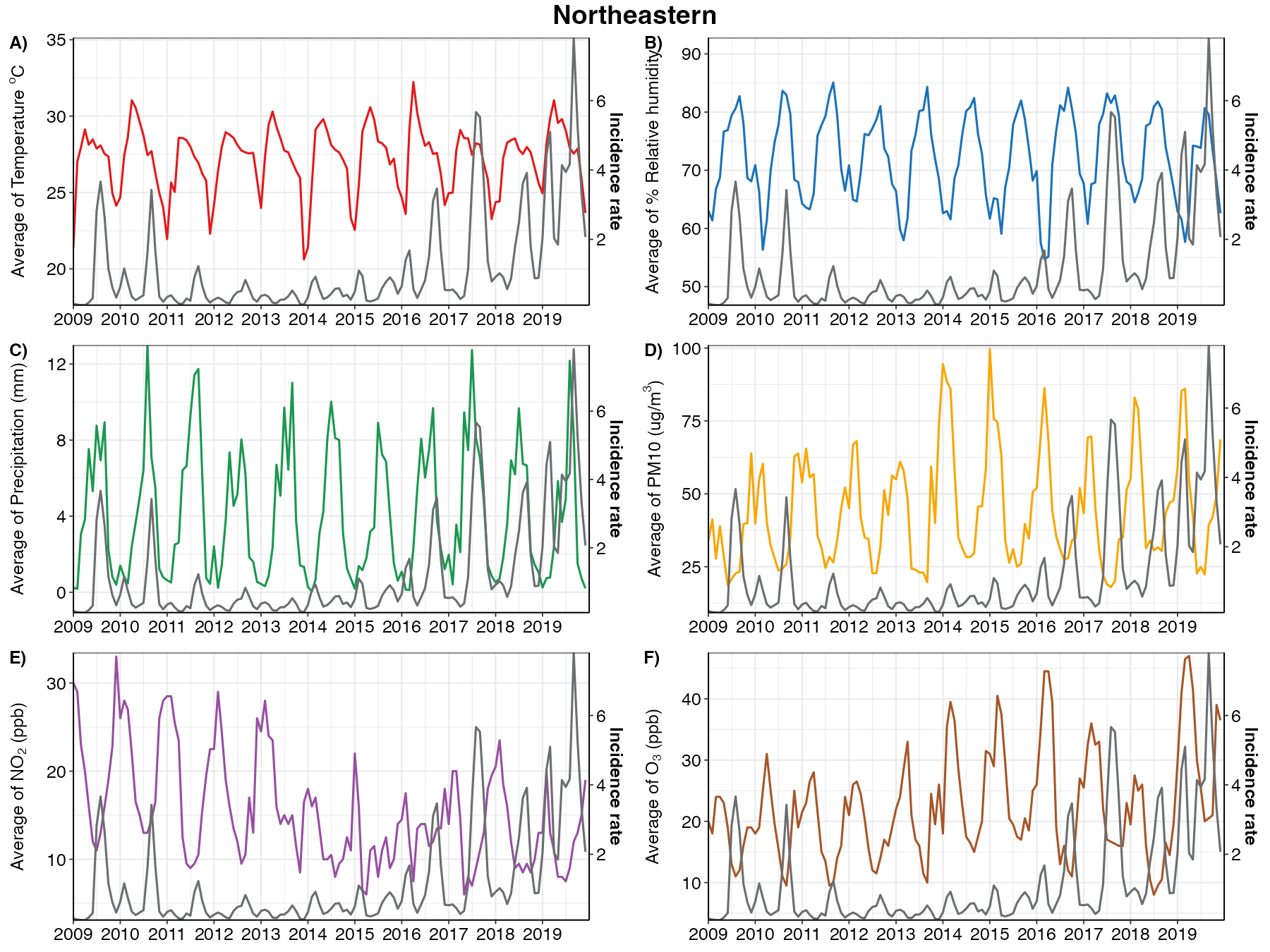
**

**Figure S2:** The time series of monthly influenza rate (cases per 10,000 population), showing in the grey color lines, combining with average temperature (^o^C) (red), average relative humidity (%) (blue), precipitation (mm) (green), PM10 (mg/m^3^) (yellow), NO_2_ (ppb) (purple), and O_3_ (ppb) (brown) in **Northeastern** region of Thailand during 2009-2019.

**
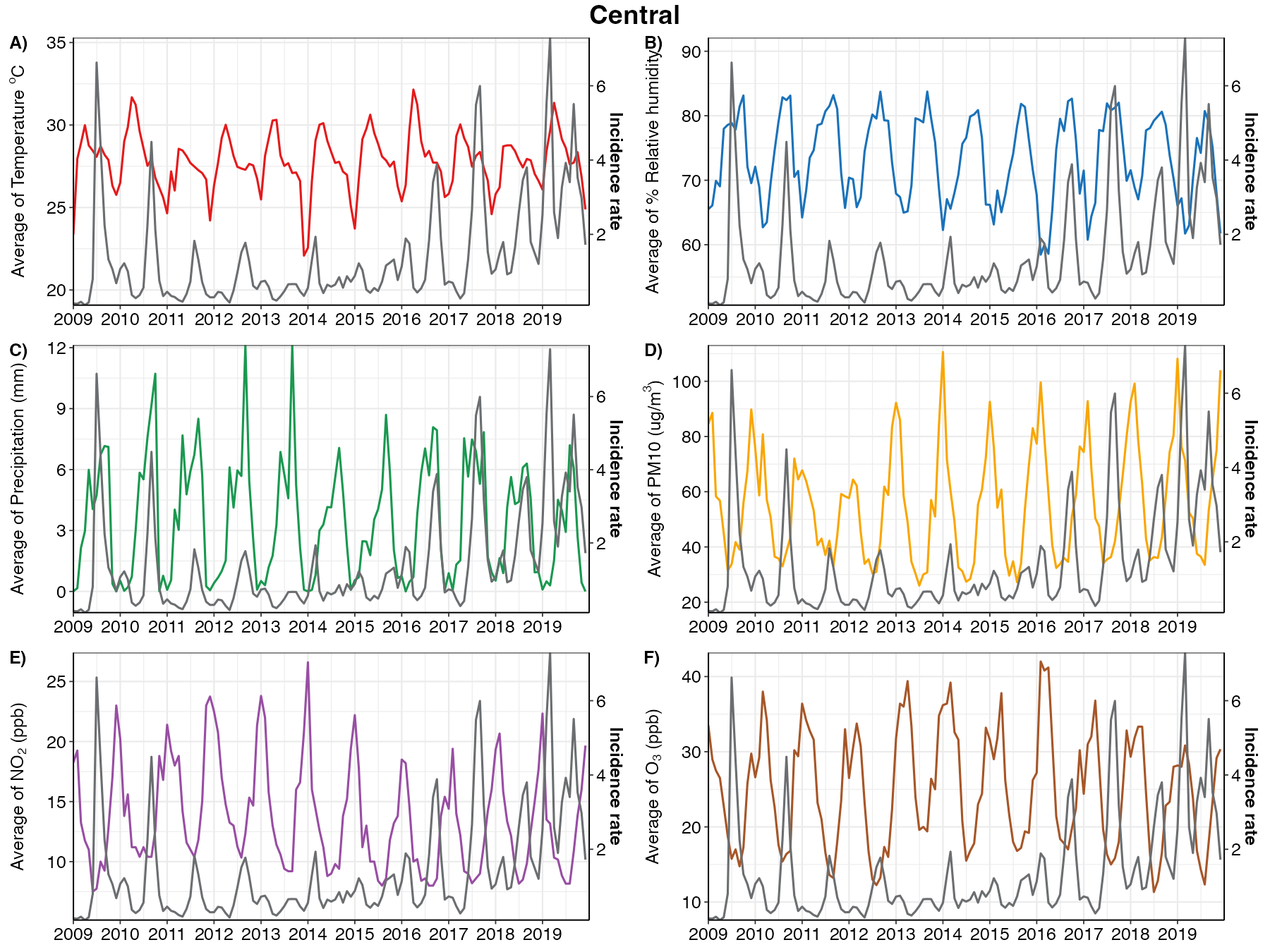
**

**Figure S3:** The time series of monthly influenza rate (cases per 10,000 population), showing in the grey color lines, combining with average temperature (^o^C) (red), average relative humidity (%) (blue), precipitation (mm) (green), PM10 (mg/m^3^) (yellow), NO_2_ (ppb) (purple), and O_3_ (ppb) (brown) in **Central** region of Thailand during 2009-2019.

**
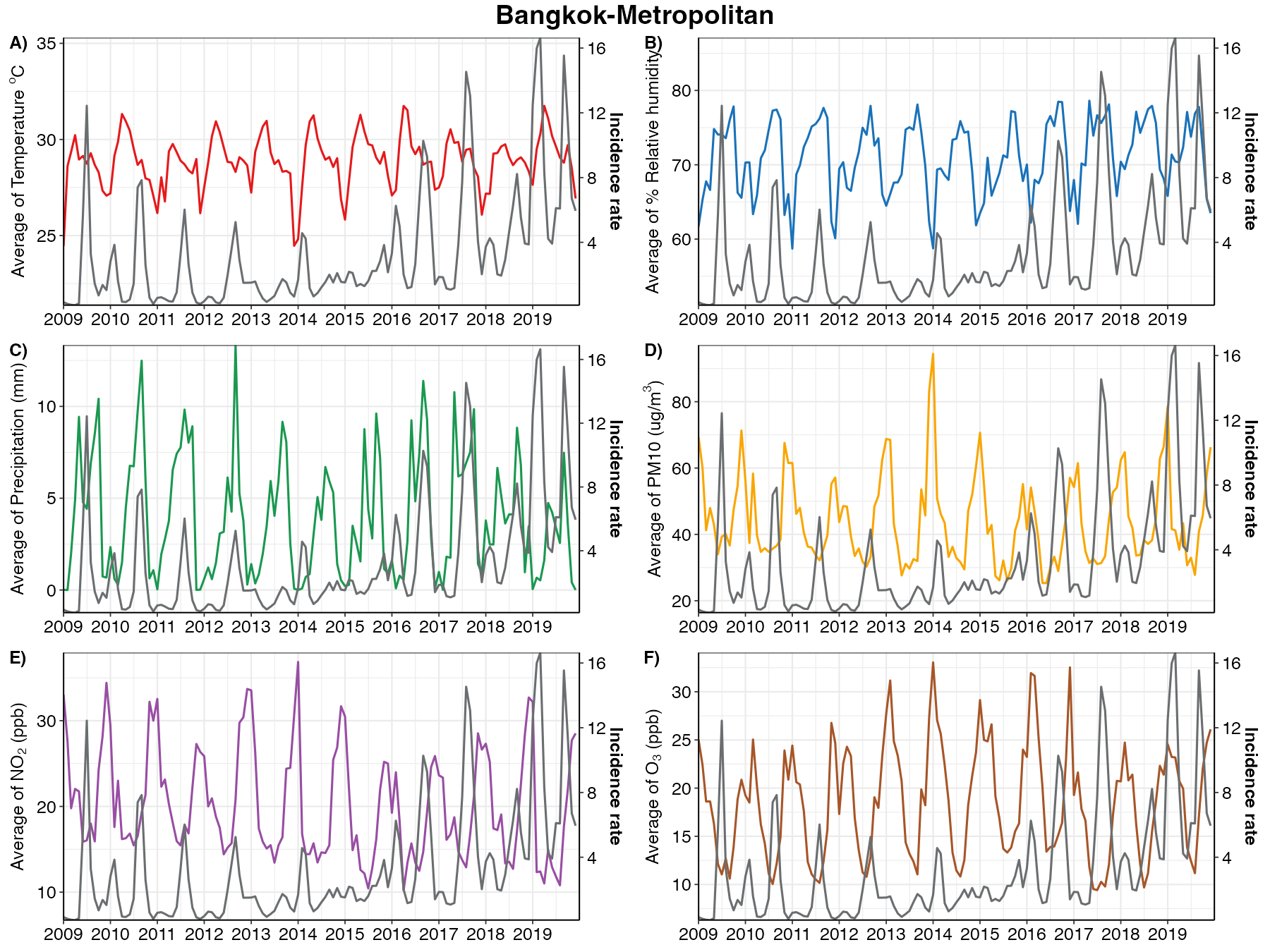
**

**Figure S4:** The time series of monthly influenza rate (cases per 10,000 population), showing in the grey color lines, combining with average temperature (^o^C) (red), average relative humidity (%) (blue), precipitation (mm) (green), PM10 (mg/m^3^) (yellow), NO_2_ (ppb) (purple), and O_3_ (ppb) (brown) in **Bangkok Metropolitan** region of Thailand during 2009-2019.

**
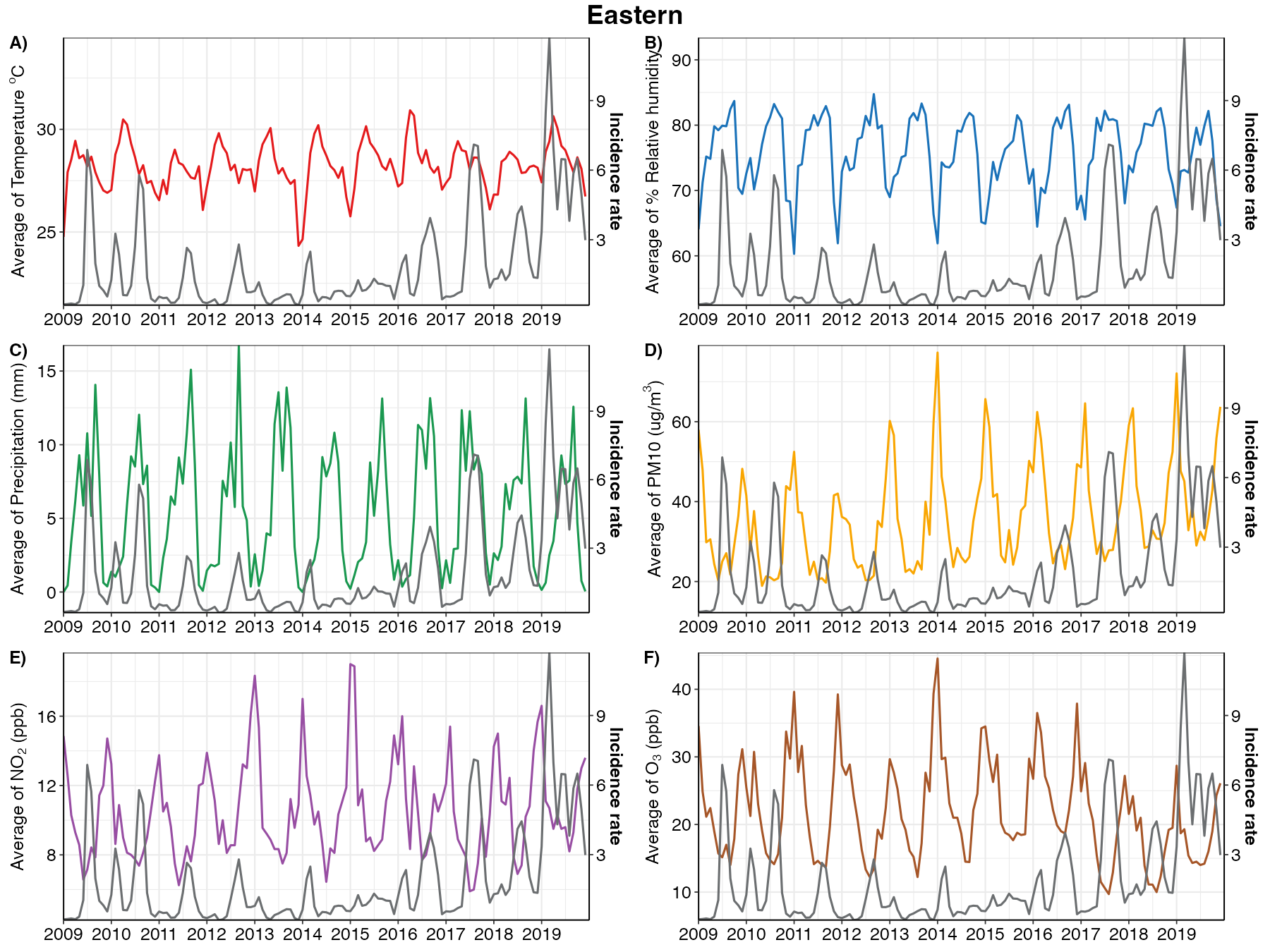
**

**Figure S5:** The time series of monthly influenza rate (cases per 10,000 population), showing in the grey color lines, combining with average temperature (^o^C) (red), average relative humidity (%) (blue), precipitation (mm) (green), PM10 (mg/m^3^) (yellow), NO_2_ (ppb) (purple), and O_3_ (ppb) (brown) in **Eastern** region of Thailand during 2009-2019.

**
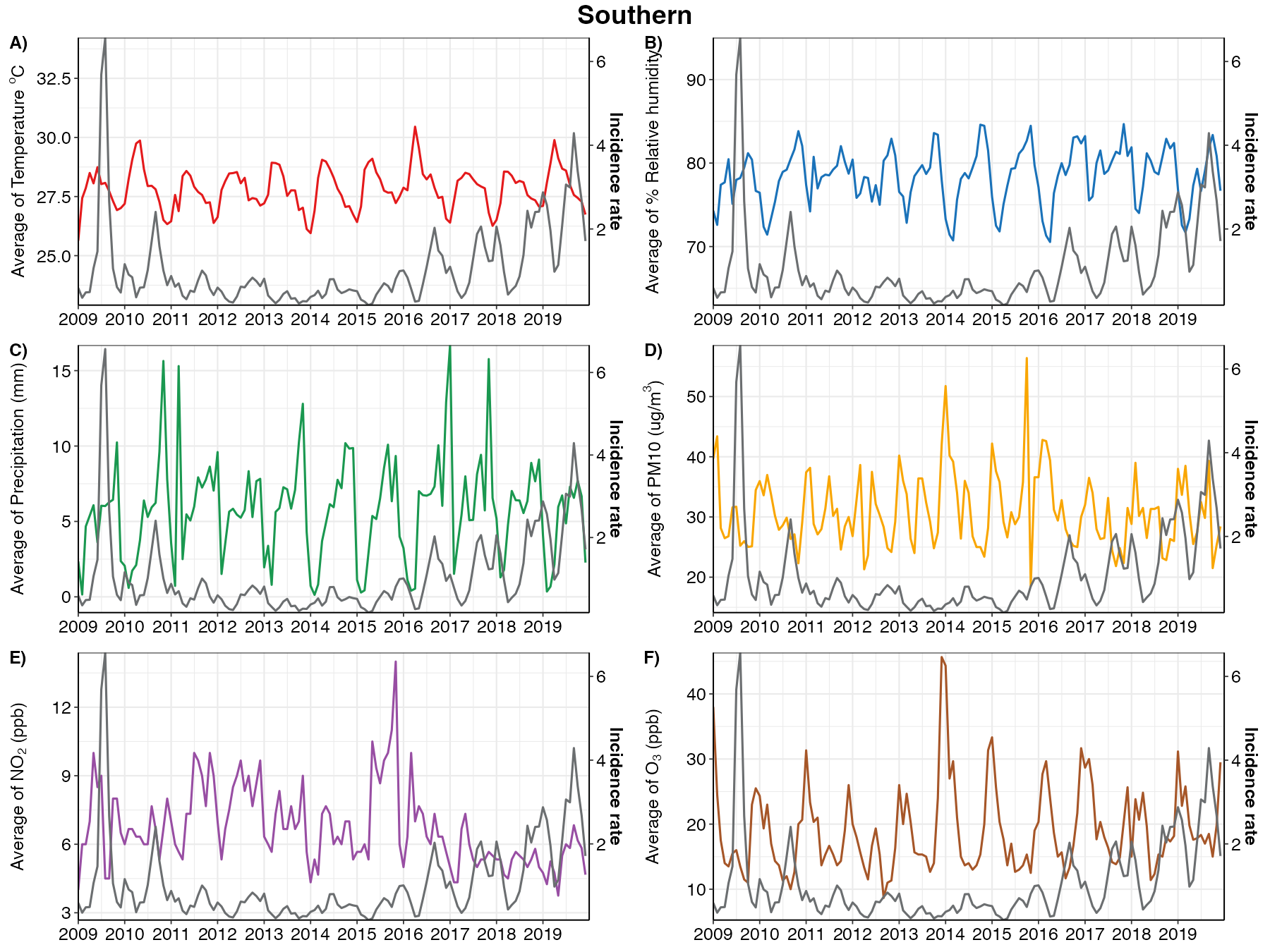
**

**Figure S6:** The time series of monthly influenza rate (cases per 10,000 population), showing in the grey color lines, combining with average temperature (^o^C) (red), average relative humidity (%) (blue), precipitation (mm) (green), PM10 (mg/m^3^) (yellow), NO_2_ (ppb) (purple), and O_3_ (ppb) (brown) in **Southern** region of Thailand during 2009-2019.


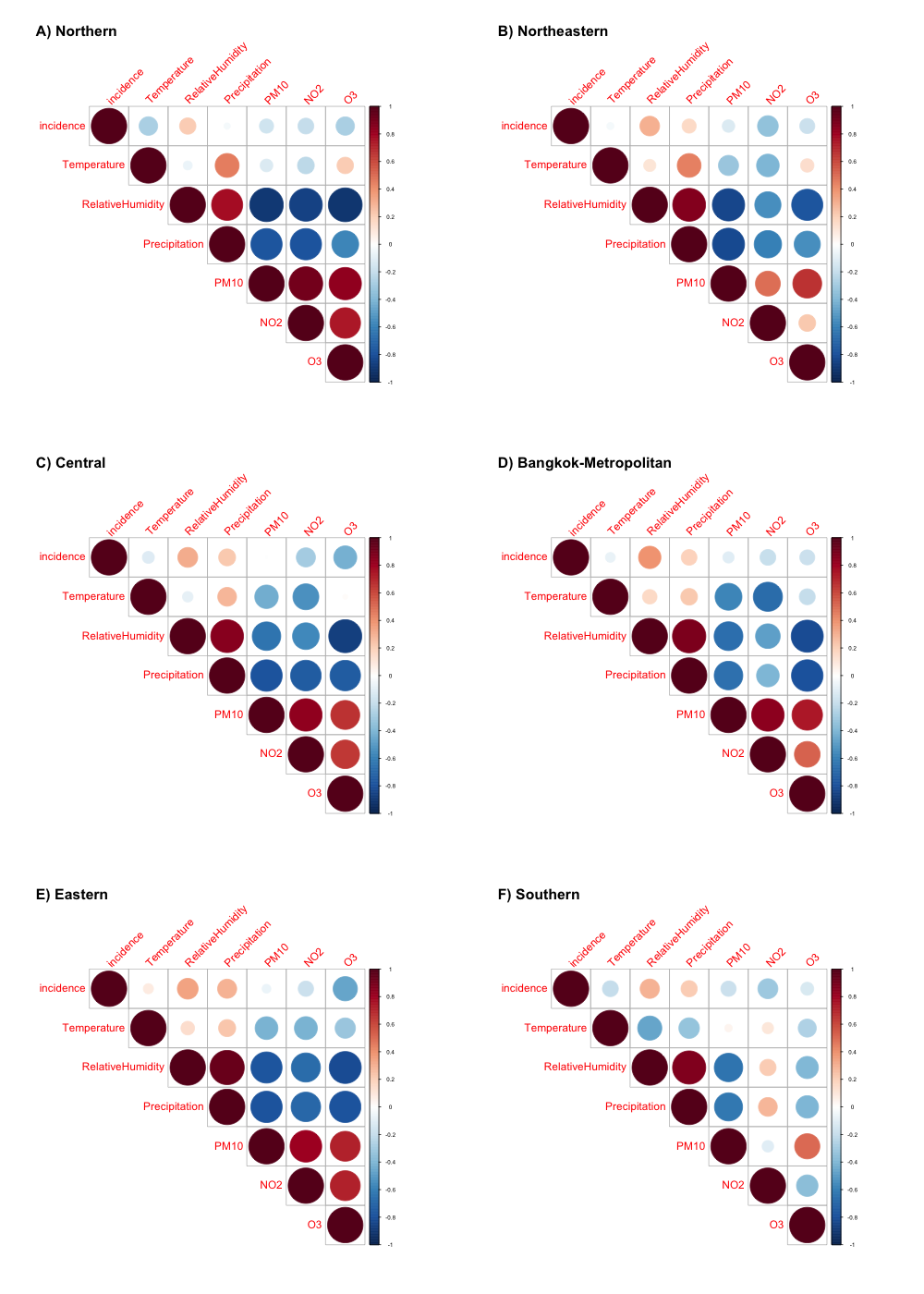


**Figure S7**: The correction coefficient between influenza incidence rate (per 10,000 population), average temperature (^o^C), average relative humidity (%), precipitation (mm), PM10 (mg/m^3^), NO_2_ (ppb), and O_3_ (ppb) for 6 regions.


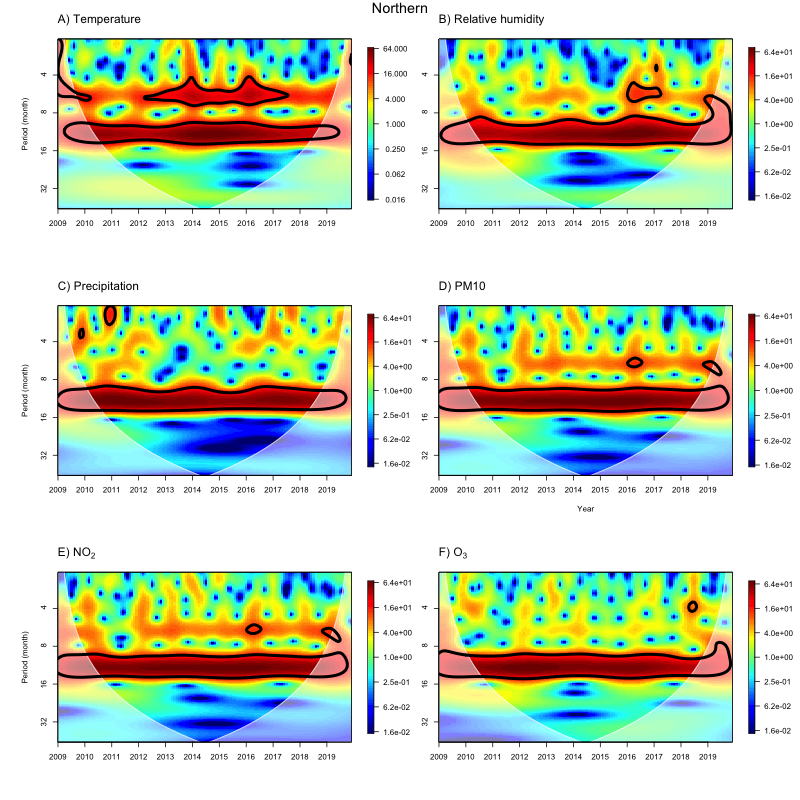


**Figure S8:** CWT spectra of temperature, relative humidity, precipitations, PM10, NO_2_, and O_3_ in northern region from the year 2009 to 2019.


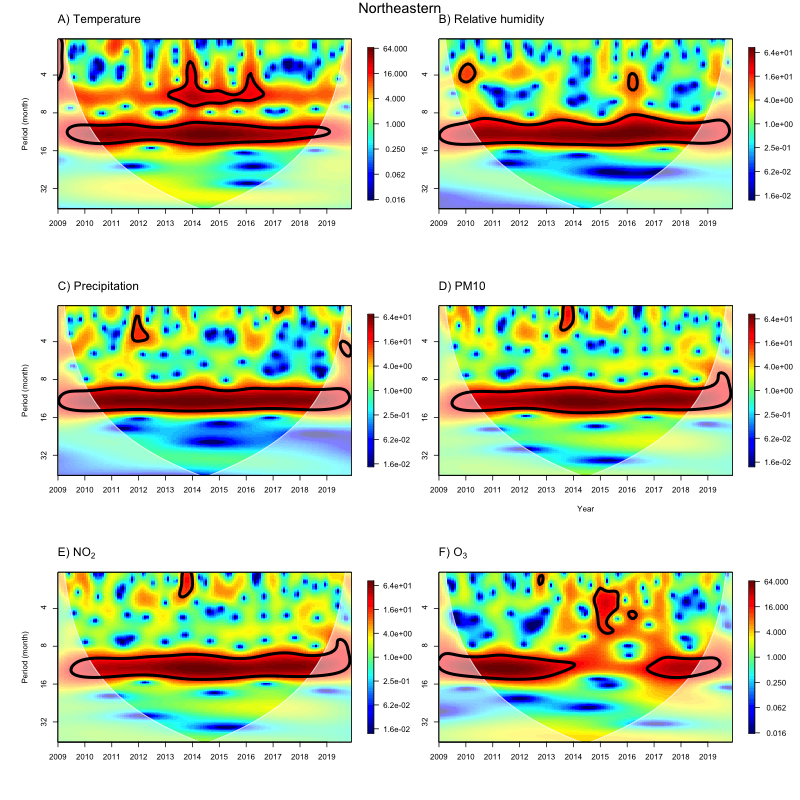


**Figure S9:** CWT spectra of temperature, relative humidity, precipitations, PM10, NO_2_, and O_3_ in northeastern region from the year 2009 to 2019.


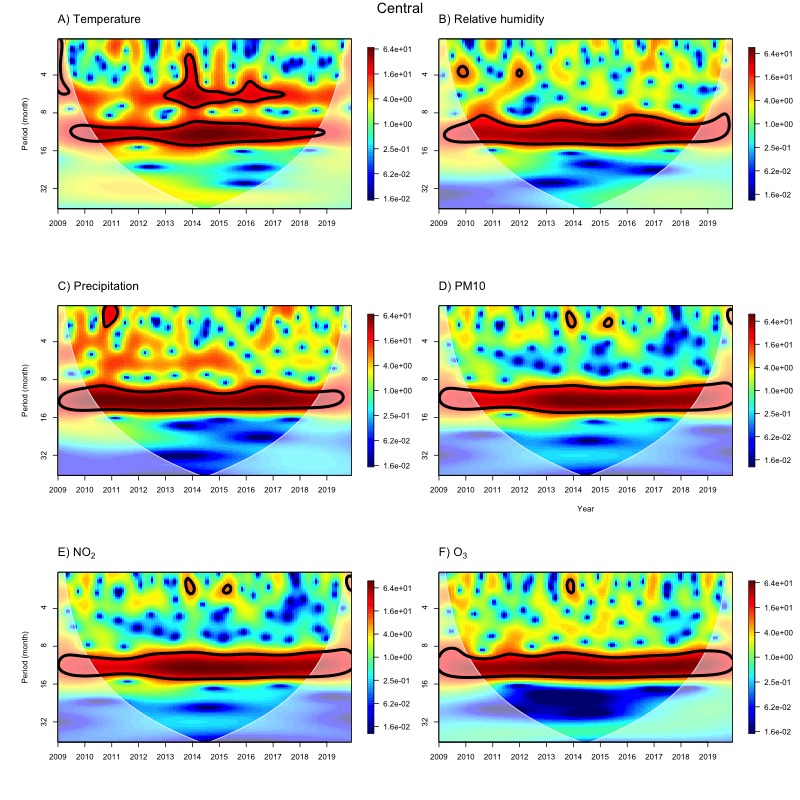


**Figure S10:** CWT spectra of temperature, relative humidity, precipitations, PM10, NO_2_, and O_3_ in central region from the year 2009 to 2019.


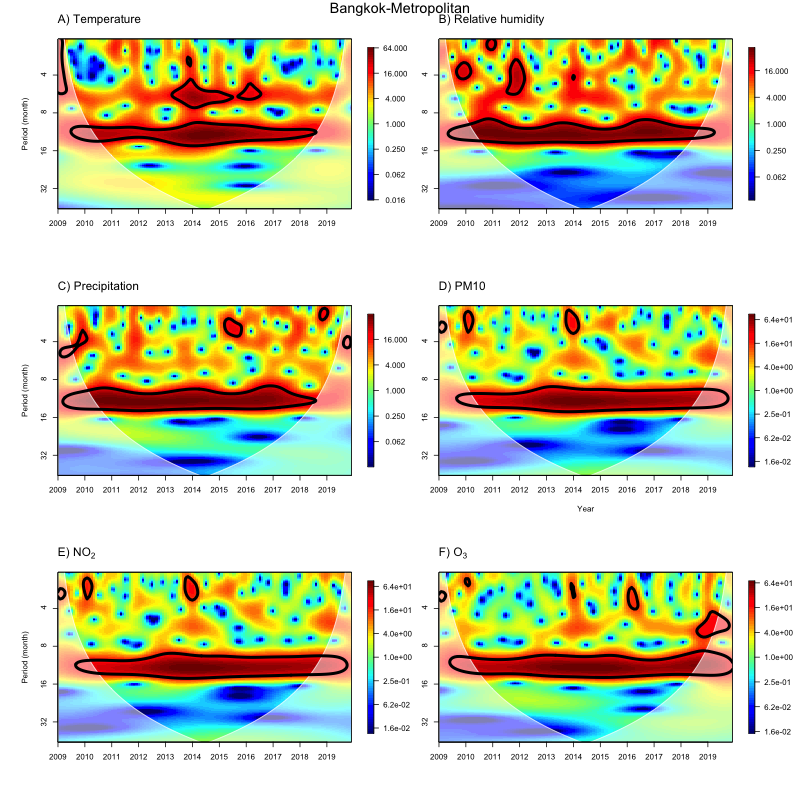


**Figure S11:** CWT spectra of temperature, relative humidity, precipitations, PM10, NO_2_, and O_3_ in Bangkok-metropolitan region from the year 2009 to 2019.


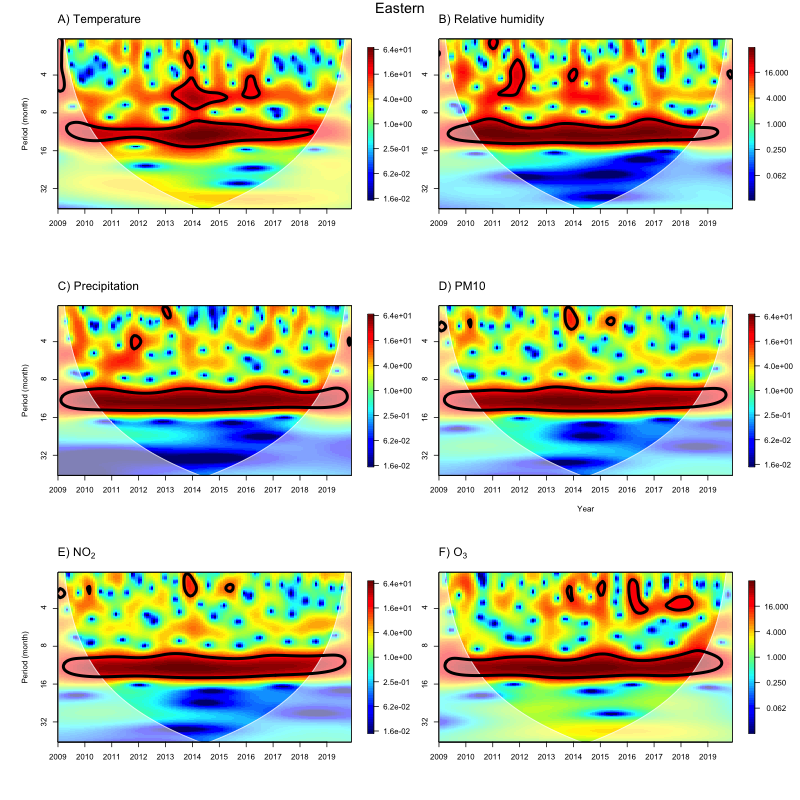


**Figure S12:** CWT spectra of temperature, relative humidity, precipitations, PM10, NO_2_, and O_3_ in eastern region from the year 2009 to 2019.


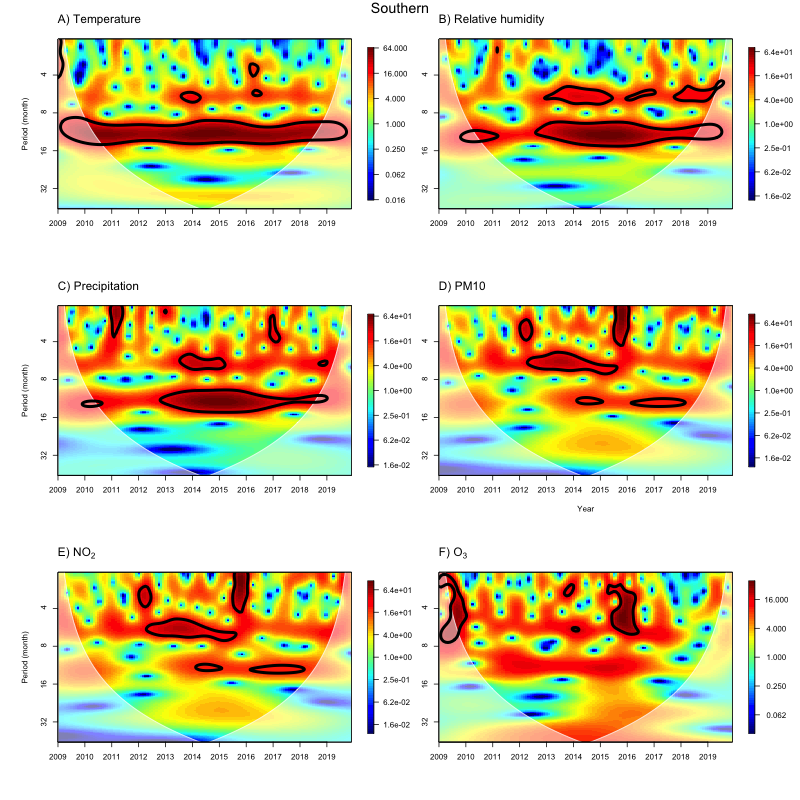


**Figure S13:** CWT spectra of temperature, relative humidity, precipitations, PM10, NO_2_, and O_3_ in southern region from the year 2009 to 2019.

**Figure S14:** Phase difference of influenza incidence with the exposure variables average at a period of 6 months for six different regions. The dots show only the significant phase difference at that particular time, corresponding with the WTC in Figure 3-8.

**Figure S15:** Phase difference of influenza incidence with the exposure variables average at a period of 12 months for six different regions. The dots show only the significant phase difference at that particular time, corresponding with the WTC in Figure 3-8.

**
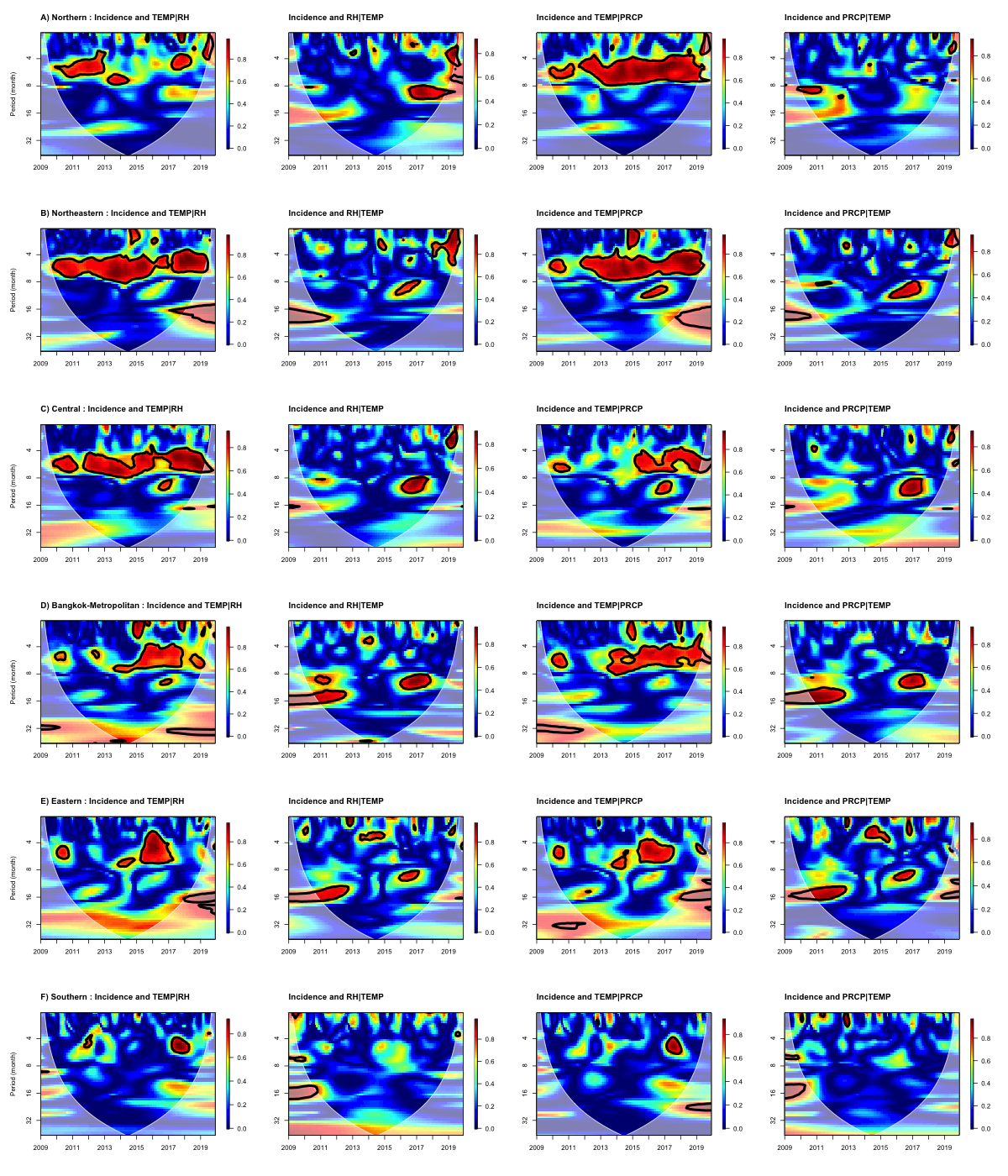
**

**Figure S16:** Partial wavelet coherence (PWC) for influenza incidence with the meteorological factors: TEMP (temperature), RH (Relative humidity), and PRCP (precipitation) for 6 regions of Thailand. The first column shows the correlation between incidence and temperature by eliminating the effect of relative humidity and vice versus in the second column. The third and the fourth column show the effect of temperature without precipitation and vice versus.


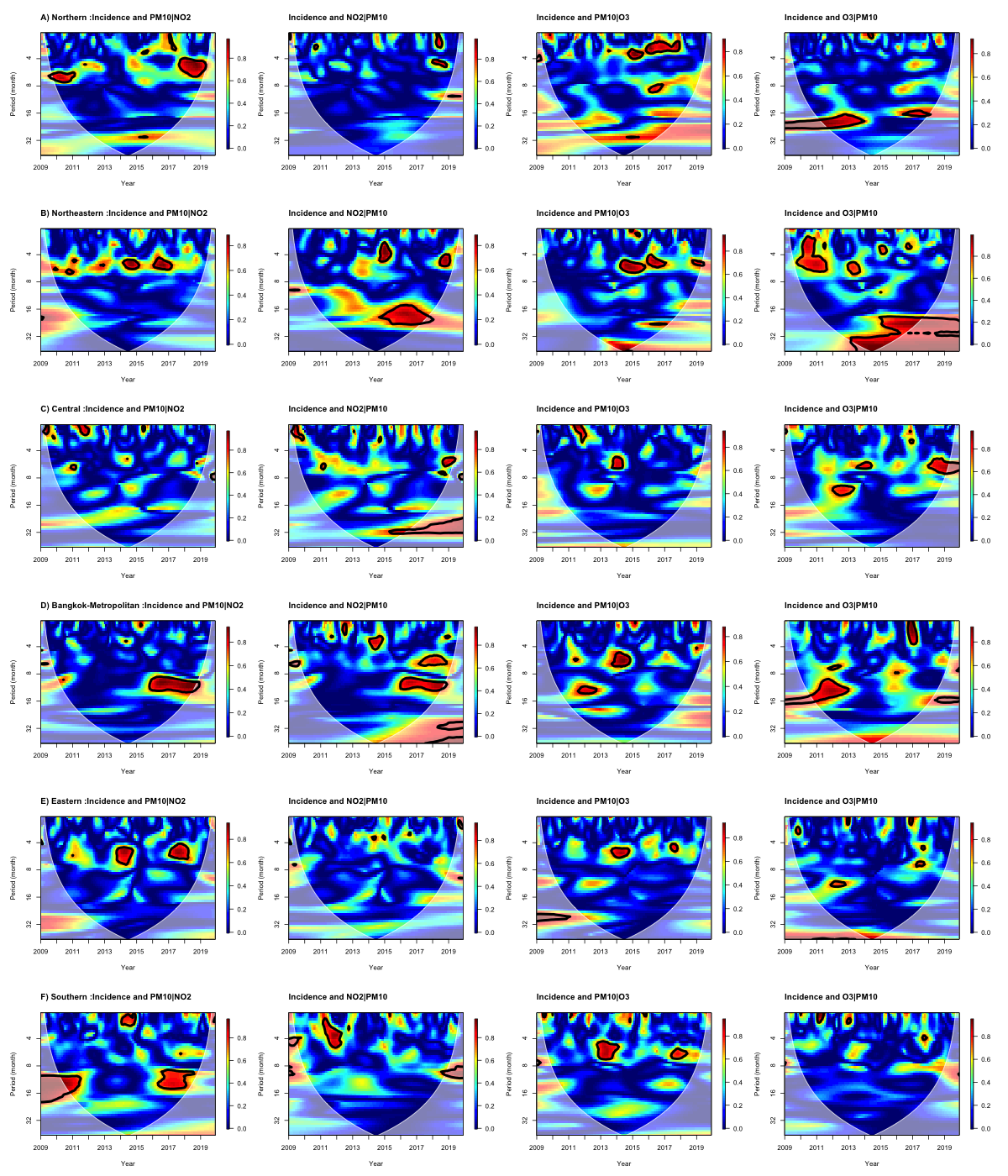


**Figure S17:** Partial wavelet coherence (PWC) for influenza incidence with the air pollution for 6 regions of Thailand. The first column shows the correlation between incidence and PM10 concentration by eliminating the effect of NO_2_ concentration and vice versus in the second column. The third and the fourth column show the effect of PM10 concentrations without O_3_ concentration and vice versus.


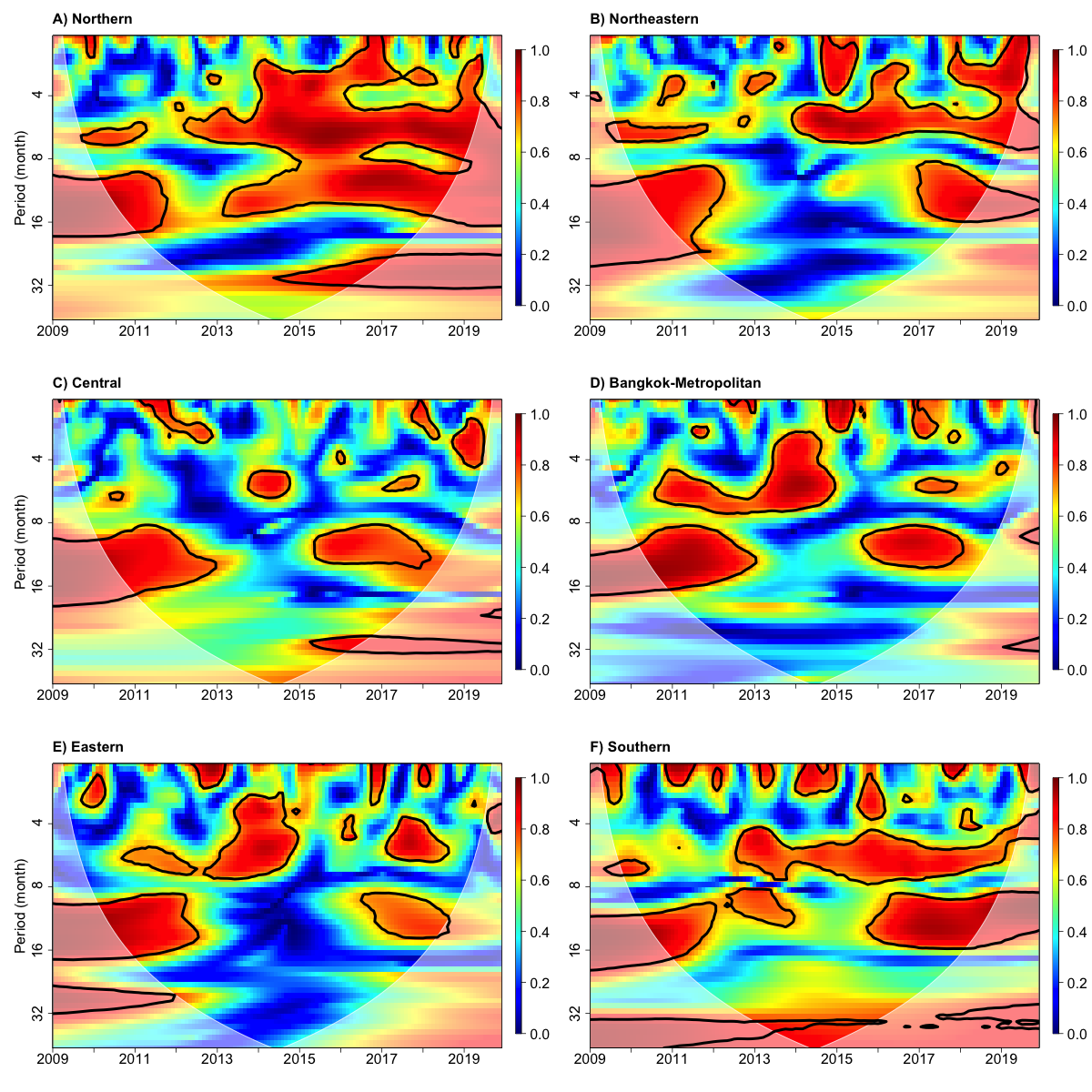


**Figure S18:** Multiple wavelet coherence (MWC) of incidence rate correlating with the linear combination of relative humidity and PM10 concentration for 6 regions of Thailand. A colored contour shows the multiple wavelet coherence, which the red (blue) color is represented the high (low) correlation in the time-period domain (with time on the x-axis and period on the y-axis). The scales matching with the color are on the right-hand side of the graph. A thick black curve shows the 5% significant level against red noise. The lighter shade area represents the cone of influence (COI) indicating the area affected by edge effects.
